# Optimized derivation and culture system of human naïve pluripotent stem cells with enhanced DNA methylation status and genomic stability

**DOI:** 10.1093/procel/pwaf053

**Published:** 2025-09-09

**Authors:** Yan Bi, Jindian Hu, Tao Wu, Zhaohui Ouyang, Tan Lin, Jiaxing Sun, Xinbao Zhang, Xiaoyu Xu, Hong Wang, Ke Wei, Shaorong Gao, Yixuan Wang

**Affiliations:** Shanghai Key Laboratory of Maternal and Fetal Medicine, Clinical and Translational Research Center of Shanghai First Maternity and Infant Hospital, School of Life Sciences and Technology, Tongji University, Shanghai 200092, China; Institute for Regenerative Medicine, State Key Laboratory of Cardiology and Medical Innovation Center, Shanghai Institute of Stem Cell Research and Clinical Translation, Shanghai East Hospital, School of Life Sciences and Technology, Tongji University, Shanghai 200120, China; Frontier Science Center for Stem Cell Research, Tongji University, Shanghai 200092, China; Shanghai Key Laboratory of Maternal and Fetal Medicine, Clinical and Translational Research Center of Shanghai First Maternity and Infant Hospital, School of Life Sciences and Technology, Tongji University, Shanghai 200092, China; Institute for Regenerative Medicine, State Key Laboratory of Cardiology and Medical Innovation Center, Shanghai Institute of Stem Cell Research and Clinical Translation, Shanghai East Hospital, School of Life Sciences and Technology, Tongji University, Shanghai 200120, China; Frontier Science Center for Stem Cell Research, Tongji University, Shanghai 200092, China; Shanghai Key Laboratory of Maternal and Fetal Medicine, Clinical and Translational Research Center of Shanghai First Maternity and Infant Hospital, School of Life Sciences and Technology, Tongji University, Shanghai 200092, China; Institute for Regenerative Medicine, State Key Laboratory of Cardiology and Medical Innovation Center, Shanghai Institute of Stem Cell Research and Clinical Translation, Shanghai East Hospital, School of Life Sciences and Technology, Tongji University, Shanghai 200120, China; Frontier Science Center for Stem Cell Research, Tongji University, Shanghai 200092, China; Shanghai Key Laboratory of Maternal and Fetal Medicine, Clinical and Translational Research Center of Shanghai First Maternity and Infant Hospital, School of Life Sciences and Technology, Tongji University, Shanghai 200092, China; Institute for Regenerative Medicine, State Key Laboratory of Cardiology and Medical Innovation Center, Shanghai Institute of Stem Cell Research and Clinical Translation, Shanghai East Hospital, School of Life Sciences and Technology, Tongji University, Shanghai 200120, China; Frontier Science Center for Stem Cell Research, Tongji University, Shanghai 200092, China; Shanghai Key Laboratory of Maternal and Fetal Medicine, Clinical and Translational Research Center of Shanghai First Maternity and Infant Hospital, School of Life Sciences and Technology, Tongji University, Shanghai 200092, China; Frontier Science Center for Stem Cell Research, Tongji University, Shanghai 200092, China; Shanghai Key Laboratory of Maternal and Fetal Medicine, Clinical and Translational Research Center of Shanghai First Maternity and Infant Hospital, School of Life Sciences and Technology, Tongji University, Shanghai 200092, China; Frontier Science Center for Stem Cell Research, Tongji University, Shanghai 200092, China; Shanghai Key Laboratory of Maternal and Fetal Medicine, Clinical and Translational Research Center of Shanghai First Maternity and Infant Hospital, School of Life Sciences and Technology, Tongji University, Shanghai 200092, China; Frontier Science Center for Stem Cell Research, Tongji University, Shanghai 200092, China; Shanghai Key Laboratory of Maternal and Fetal Medicine, Clinical and Translational Research Center of Shanghai First Maternity and Infant Hospital, School of Life Sciences and Technology, Tongji University, Shanghai 200092, China; Frontier Science Center for Stem Cell Research, Tongji University, Shanghai 200092, China; Shanghai Key Laboratory of Maternal and Fetal Medicine, Clinical and Translational Research Center of Shanghai First Maternity and Infant Hospital, School of Life Sciences and Technology, Tongji University, Shanghai 200092, China; Institute for Regenerative Medicine, State Key Laboratory of Cardiology and Medical Innovation Center, Shanghai Institute of Stem Cell Research and Clinical Translation, Shanghai East Hospital, School of Life Sciences and Technology, Tongji University, Shanghai 200120, China; Frontier Science Center for Stem Cell Research, Tongji University, Shanghai 200092, China; Institute for Regenerative Medicine, State Key Laboratory of Cardiology and Medical Innovation Center, Shanghai Institute of Stem Cell Research and Clinical Translation, Shanghai East Hospital, School of Life Sciences and Technology, Tongji University, Shanghai 200120, China; Frontier Science Center for Stem Cell Research, Tongji University, Shanghai 200092, China; Shanghai Key Laboratory of Maternal and Fetal Medicine, Clinical and Translational Research Center of Shanghai First Maternity and Infant Hospital, School of Life Sciences and Technology, Tongji University, Shanghai 200092, China; Institute for Regenerative Medicine, State Key Laboratory of Cardiology and Medical Innovation Center, Shanghai Institute of Stem Cell Research and Clinical Translation, Shanghai East Hospital, School of Life Sciences and Technology, Tongji University, Shanghai 200120, China; Frontier Science Center for Stem Cell Research, Tongji University, Shanghai 200092, China; Shanghai Key Laboratory of Maternal and Fetal Medicine, Clinical and Translational Research Center of Shanghai First Maternity and Infant Hospital, School of Life Sciences and Technology, Tongji University, Shanghai 200092, China; Institute for Regenerative Medicine, State Key Laboratory of Cardiology and Medical Innovation Center, Shanghai Institute of Stem Cell Research and Clinical Translation, Shanghai East Hospital, School of Life Sciences and Technology, Tongji University, Shanghai 200120, China; Frontier Science Center for Stem Cell Research, Tongji University, Shanghai 200092, China

**Keywords:** human naïve culture system, bifluorescence reporter system, high-content analysis, DNA methylation, genomic stability

## Abstract

Human naïve pluripotent stem cells (PSCs) hold great promise for embryonic development studies. Existing induction and culture strategies for these cells, heavily dependent on MEK inhibitors, lead to widespread DNA hypomethylation, aberrant imprinting loss, and genomic instability during extended culture. Here, employing high-content analysis alongside a bifluorescence reporter system indicative of human naïve pluripotency, we screened over 1,600 chemicals and identified seven promising candidates. From these, we developed four optimized media—LAY, LADY, LUDY, and LKPY—that effectively induce and sustain PSCs in the naïve state. Notably, cells reset or cultured in these media, especially in the LAY system, demonstrate improved genome-wide DNA methylation status closely resembling that of pre-implantation counterparts, with partially restored imprinting and significantly enhanced genomic stability. Overall, our study contributes advancements to naïve pluripotency induction and long-term maintenance, providing insights for further applications of naïve PSCs.

## Introduction

Pre-implantation epiblast cells, existing in the naïve or ground pluripotent state, represent a higher developmental hierarchy position and demonstrate greater differentiation potentials compared to post-implantation epiblast cells in the primed state. *In vitro* models of human naïve pluripotent stem cells (PSCs), achieved by direct derivation from pre-implantation embryos, somatic reprogramming, or conversion from primed PSCs using chemical and genetic approaches. Female naïve PSCs display an X chromosome state resembling that of pre-implantation blastocysts, offering a valuable model for investigating X chromosome regulation in human cells. Recent breakthroughs have demonstrated that human naïve PSCs can differentiate into trophectoderm (Guo et al., 2021; Io et al., 2021) and primitive endoderm lineages (Linneberg-Agerholm et al., 2019). Moreover, human naïve PSCs possess the potential to form a blastoid through serial differentiation induction (Kagawa et al., 2022; Yanagida et al., 2021; Yu et al., 2021, 2023), making them invaluable tools for peri-implantation development studies and clinical research applications.

*In vitro* capture of human naïve state was inspired by mouse studies based on the use of the MEK inhibitor PD0325901, GSK3 inhibitor CHIR99021, and cytokine LIF (2i/L) (Hanna et al., 2010). Various naïve culture conditions, including 3i/L (Chan et al., 2013), NHSM (Gafni et al., 2013), t2iLGö (Takashima et al., 2014), 5iLAF (Theunissen et al., 2014), PXGL (Bredenkamp et al., 2019; Guo et al., 2017), HENSM (Bayerl et al., 2021), and 4CL (Mazid et al., 2022), facilitate the acquisition and maintenance of a naïve-like state in human PSCs. Notably, among these conditions, t2iLGö (2iL supplemented with the protein kinase K inhibitor Gö6893, along with transient overexpression of NANOG and KLF2) (Takashima et al., 2014), 5iLAF (2iL supplemented with inhibitors targeting MEK, GSK3, RAF, SRC, and ROCK, in addition to bFGF and activin A) (Theunissen et al., 2014), and PXGL (2iLGö modified with XAV939) represent widely recognized naïve culture systems (Bredenkamp et al., 2019; Guo et al., 2017). These closely mirror the transcriptomic profile of pre-implantation naïve epiblasts and display similarities to pre-implantation blastocysts.

Long-term usage of 2i, especially MEK inhibitor (MEKi), in naïve PSC culture systems, has been reported to induce irreversible changes, leading to significant loss of DNA methylation (Theunissen et al., 2016), extensive erasure of genomic imprints (Choi et al., 2017), and compromised genome integrity (Di Stefano et al., 2018). Reducing the dosage of MEK inhibitors can partially mitigate these deleterious effects, thus improving genomic stability in human naïve embryonic stem cells (ESCs) (Di Stefano et al., 2018). However, even under optimized protocols such as t2iLGö or 5iLAF by which naïve ESCs can be efficiently derived from blastocysts, these cell lines still display DNA hypomethylation compared to pre-implantation epiblasts (Pastor et al., 2016). Importantly, such DNA methylation defects in naïve PSCs jeopardize genomic stability (Di Stefano et al., 2018; Liu et al., 2017), highlighting the need for further optimization of naïve state PSC culture conditions.

In this study, using the ALPG-promoter-RFP; OCT4-△PE-GFP bifluorescence reporter system we established before, we screened seven chemicals in the absence of 2i by high-content analysis (HCA) using over 1,600 well-annotated chemicals library. Through systematic combinations, we successfully identified four optimal media, LAY, LADY, LUDY, and LKPY, that demonstrate broad applicability and robustness in both the induction and maintenance of human PSCs in the naïve pluripotent state, corresponding to the pre-implantation stage *in vivo*. Notably, we comprehensively compared existing human naïve PSC culture systems in terms of whole-genome DNA methylation levels and found that the newly developed LAY system achieves optimized genome-wide DNA methylation levels, addressing the abnormality of hypomethylation caused by long-term culture in the presence of 2i in current systems, and thus significantly enhancing genomic stability of naïve PSCs. Taken together, our study provides significant refinements for naïve pluripotency induction and long-term maintenance, offering new insights for further applications of naïve PSCs.

## Results

### High-content analysis screening to identify chemicals important for human naïve pluripotency in a naïve-primed-naïve induction system with a dual reporter system

To identify potential alternative chemicals important for naïve pluripotency establishment, we first set up a chemical screening system on a high-throughput scale by adopting the ALPG-promoter-RFP; OCT4-ΔPE-GFP bifluorescence reporter system that developed previously to precisely delineate the transition into and out of the naïve pluripotent state (Bi et al., 2020, 2022). To determine the timepoints for screen monitoring, we developed a naïve-to-primed-to-naïve transition system (abbreviated as npn transition system). Specifically, we first seeded RFP^+^GFP^+^ naïve PSCs on feeder layers in 5iLAF media. Subsequently, we transitioned to the conventional primed PSCs culture medium for 48 h, observing a majority of cells ephemerally became RFP^−^GFP^−^, which suggests a temporarily primed-like state of these cells. Upon transferring these primed-like cells back to 5iLAF media and culturing them 96 h, 50.5% reverted to RFP^+^GFP^+^ cells, re-acquiring the naïve state (Fig. S1A). Leveraging this npn transition system, the efficacy of chemicals in facilitating naïve pluripotency can be rapidly ascertained.

**Figure 1.**
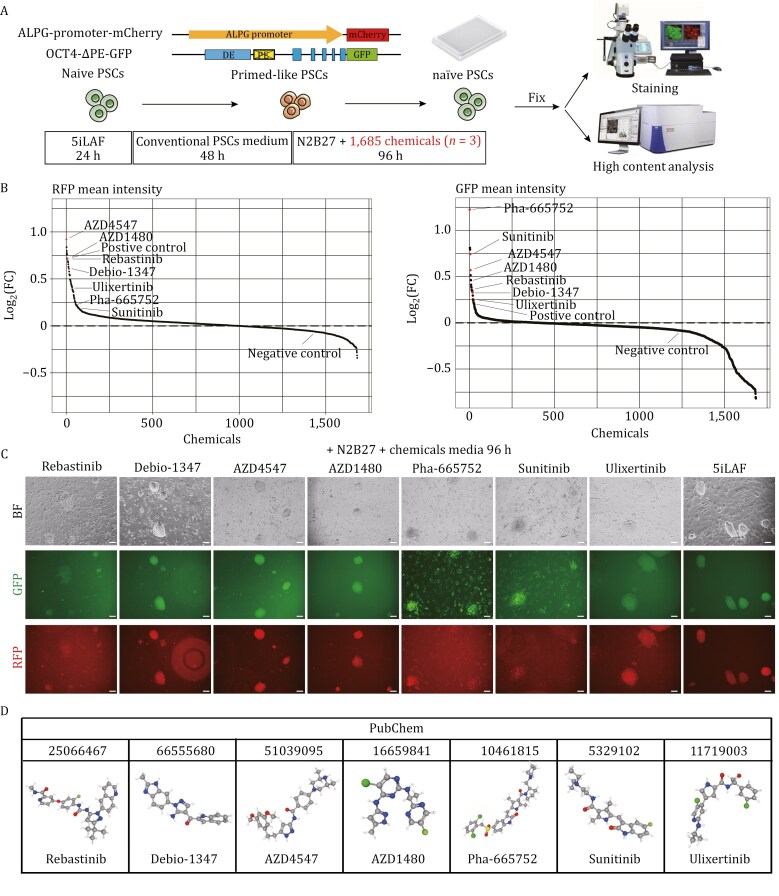
Identiﬁcation of seven key chemicals enhancing human naïve pluripotency within a naïve-primed-naïve (npn) transition system through high-content screening. (A) Schematic overview of the npn transition system with a bifluorescence reporter indicative of human naïve pluripotency, facilitating the high-content screening of important chemicals that promote naïve pluripotency. (B) Scatter plots illustrating the average fluorescence intensities of RFP and GFP signals for 1,685 compounds assessed in the npn transition system, relative to a negative control. Note that the seven chemicals of interest, along with the positive control, are ranked in the top 78 and top 25 for RFP and GFP. (C) Bioﬂuorescence imaging showcases the effects of the seven selected chemicals and 5iLAF media on the npn transition system. Scale bars, 100 µm. (D) PubChem identifiers and names of the seven selected chemicals.

Next, we subjected the npn transition system to an inhibitor library consisting of 1,685 well-defined chemicals (Selleck, Inhibitor Library #L1100) with HCA. In our initial screening, 7 × 10^3^ RFP^+^GFP^+^ naïve PSCs seeded per well were subjected to 1 μmol/L of each screening compound in N2B27 basal media and refreshed daily, using 5iLAF media as positive controls, N2B27 basal media with 0.1% Dimethyl sulfoxide (DMSO) as standard controls, and conventional PSCs medium as negative controls. In the HCA pipeline, we employed the automated image analysis software, CellProfiler, to identify and count cell nuclei based on the 4', 6-diamidino-2-phenylindole (DAPI) channel, capture fluorescent images, and compute RFP and GFP fluorescence intensities 96 h after chemical culture (Fig. 1A).

Using this analytical pipeline, we calculated the fold changes in integrated intensities for RFP and GFP across all wells (Figs. 1B and S1B). Among the chemicals tested, 996 exhibited increased RFP^+^ ratios compared to both negative and standard control wells; these chemicals were predominantly related to pathways involving MAPK, DNA damage, and angiogenesis. Meanwhile, 404 chemicals showing heightened GFP^+^ ratios post-treatment were mainly associated with the MAPK pathway (Fig. S1C), suggesting that the MAPK pathway plays a crucial role in promoting naïve pluripotent state. After excluding chemicals that target the MEK pathway and those affecting cell proliferation, we narrowed down to seven chemicals that notably enhanced the bifluorescence ratio. These include Rebastinib (DCC-2036), an Abl1 inhibitor; Debio-1347 (Zoligratinib), an FGFR1/2/3/4 inhibitor; AZD4547 (ABSK091), a selective FGFR inhibitor; AZD1480, an ATP-competitive JAK2 inhibitor; Pha-665752, an inhibitor of c-Met, RON and Flk1; Sunitinib, targeting the VEGF and PDGF/PDGFR pathways; and Ulixertinib (BVD-523), an ERK1/2 inhibitor (Fig. 1C and 1D).

To determine the optimal concentrations of each selected chemical for cell viability and biofluorescence, we screened varying concentrations (from 0.1 to 51.2 μmol/L) of these seven chemicals in N2B27 basal media using HCA (*n* = 3). Based on this screening, we obtained specific concentrations for each chemical: 2.5 μmol/L for Rebastinib, 1.5 μmol/L for Sunitinib and Ulixertinib, 1 μmol/L for Debio-1347, AZD4547 and Pha-665752, and 0.5 μmol/L for AZD1480 (Table S1).

### Chemical combinations to establish culture systems for human naïve pluripotency sustention, resetting

**Figure 2.**
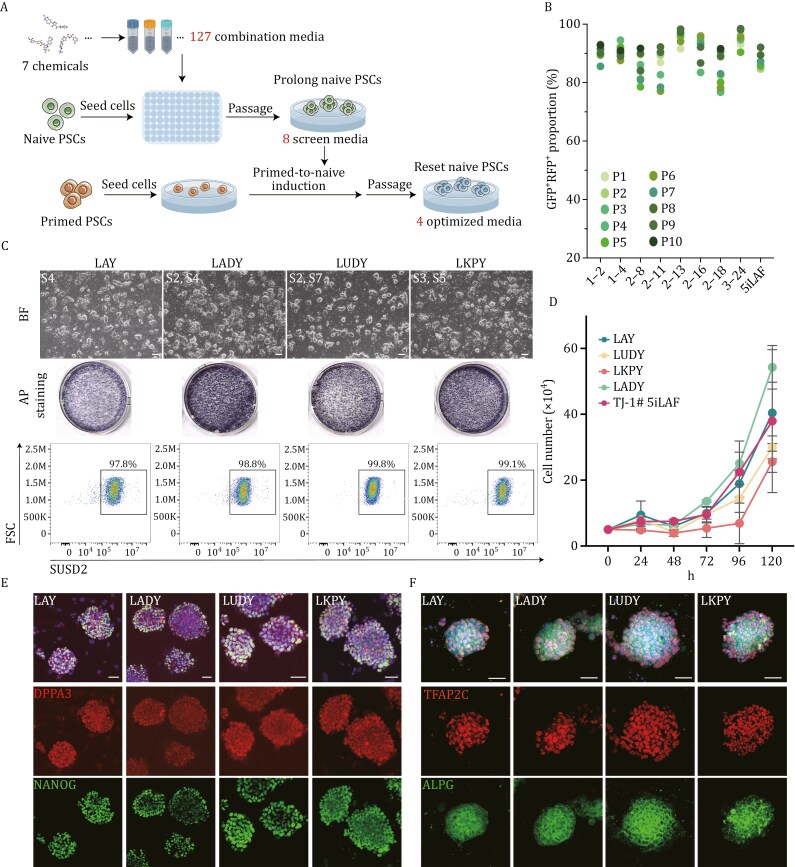
Identification and characterization of chemical combinations for naïve pluripotency maintenance and establishment. (A) Schematic representation of the process of evaluating 127 distinct chemical combinations tested for sustaining and resetting naïve PSCs using 5iLAF as the positive control. (B) Statistical analysis of the bifluorescence ratios in naïve PSCs across 10 generations under eight distinct culture conditions. (C) Representative phase images (top), alkaline phosphatase (AP) staining (middle), and flow cytometry for SUSD2 expression (bottom) in reset naïve PSCs treated with LAY, LADY, LUDY, and LKPY media at Passage 20, respectively. Scale bars, 200 µm. (D) Growth curves of naïve PSCs reset with LAY, LADY, LUDY, LKPY, and 5iLAF (*n* = 3). Error bars indicate the mean ± SD. (E and F) Immunoﬂuorescence microscopy demonstrating the expression of naïve pluripotency markers in naïve PSC reset with LAY, LADY, LUDY, and LKPY conditions. Scale bars, 50 µm.

Next, to investigate whether the synergistic effects of these seven molecules could significantly enhance and sustain naïve pluripotency, human naïve PSCs, previously reset and cultured in 5iLAF media, were seeded and subjected to 127 distinct media consisting of various combinations of the seven identified chemicals, each at its optimal concentration, in N2B27 basal media. For comparison, we included 5iLAF media as a positive control, conventional PSCs medium as a negative control, and N2B27 basal media with 0.165% DMSO as the standard control (Fig. 2A; Table S1). Most culture conditions led to cell death or differentiation. However, among 64 combinations that supported cell survival, only 28 sustained cell viability to three passages, as indicated by the maintenance of the bifluorescence ratios (Fig. S2A).

To prevent cell death and enhance the quality of naïve colonies, human LIF (L) and the ROCK inhibitor Y-27632 (Y) were supplemented into the above media for prolong culture. Subsequent re-screening identified 11 chemical combinations capable of sustaining cells with naïve clonal morphologies for over 10 generations in the N2B27/LY media. Among these, two media (1–5, 1–7) displayed low bifluorescence ratios, while one combination (2–9) impeded cellular proliferation during successive passages (Fig. S2B and S2C). Consequently, we finally identified eight effective culture conditions, which not only supported regular cell proliferation but also ensured high fluorescence ratios (RFP^+^GFP^+^% > 85%) beyond the 10th generation (Figs. 2B, S2D, and S2E).

Given the proficiency of these eight culture conditions in sustaining prolong cultures of human PSCs at naïve state, we further explored their potential in facilitating the primed-to-naïve resetting of the TJ-1# primed PSCs cell line (named as TJ-1# Primed), previously derived from a blastocyst, and the establishment of naïve state PSC lines (Fig. 2A). Among the conditions, 1–2, 2–16, 2–18, and 3–24 conditions failed to yield naïve-like cells during the primed-to-naïve transition (Fig. S3A). However, four culture media (1–4, 2–8, 2–11, and 2–13) could efficiently reset primed PSCs and establish stable naïve PSCs lines. Based on the chemicals used in their media, we named these naïve culture systems LAY, LAKY, LUDY, and LKPY (Fig. S2E). These naïve PSCs were characterized by their capacity to stably propagate for more than 20 passages, displaying typical naïve morphologies and growth rates, high SUSD2^+^ ratios (Figs. 2C, 2D and S3B), and naïve pluripotency marker profiles (Fig. 2E and 2F). Additionally, karyotyping analysis verified the preservation of a normal karyotype for up to 25 generations (Fig. S3C).

We also tested these four culture conditions for primed-to-naïve resetting using the TJ-1# primed PSCs line engineered with the dual fluorescence reporter system. Although with a slower rate of naïve resetting compared to 5iLAF induction, the majority of cells under these four conditions were able to maintain a high proportion of dual fluorescence within five passages of resetting (Fig. S3D).

In addition to PSCs, we also assessed our culture conditions using the iPSC reprogramming system. Specifically, somatic fibroblasts were transduced with lentiviruses encoding doxycycline (Dox)-inducible Yamanaka factors to generate naïve iPSC clones under the four culture conditions. The resulting naïve iPSC lines were successfully maintained for more than 15 passages in LAY, LADY, LUDY, and LKPY media and confirmed high expression of SUSD2 (Fig. S3E).

Overall, through evaluating 127 combinations of seven selected chemicals, we successfully identified four novel culture conditions—LAY, LADY, LUDY, and LKPY—that are capable of both the derivation and maintenance of naïve pluripotency.

### Global transcriptome profiling

**Figure 3.**
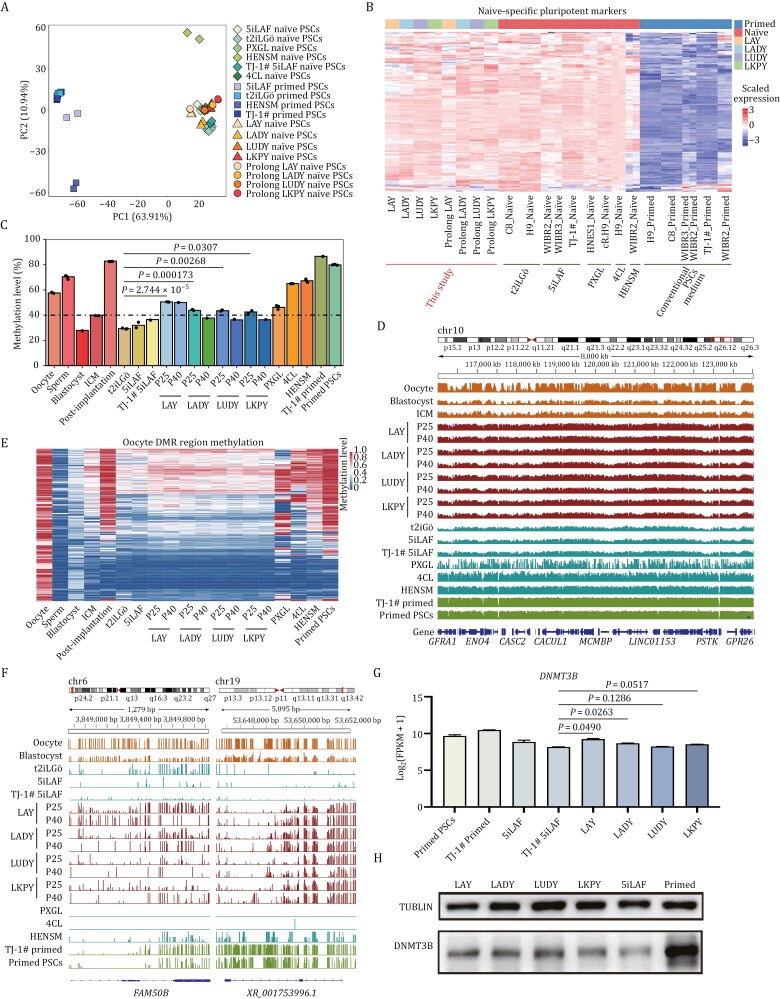
Genome-wide characterization of naïve PSCs under four defined culture conditions. (A) Principal component analysis (PCA) of the transcriptional profiles from four reset and four prolong cultures from our datasets (*n* = 2), compared with naïve and primed PSCs from published datasets. (B) Heatmap showing the expression of naive-specific pluripotent markers in the samples (*n* = 2) analyzed in (A). (C) Bar plot illustrating the average DNA methylation levels across various developmental stages and cell types, including oocyte (*n* = 2), sperm (*n* = 4), blastocyst, inner cell mass (ICM, *n* = 2), post-implantation embryos (*n* = 3), t2iLGö naïve PSCs (*n* = 3), 5iLAF naïve PSCs (*n* = 2), TJ-1# 5iLAF naïve PSCs, LAY reset PSCs (Passage 25, P25) (*n* = 2), LAY reset PSCs (Passage 40, P40), LADY reset PSCs (P25) (*n* = 2), LADY reset PSCs (P40), LUDY reset PSCs (P25) (*n* = 2), LUDY reset PSCs (P40), LKPY reset PSCs (P25) (*n* = 2), LKPY reset PSCs (P40), PXGL naïve PSCs (*n* = 3), 4CL naïve PSCs (*n* = 2), HENSM naïve PSCs (*n* = 3), TJ-1# primed PSCs, and published primed PSCs (*n* = 3). The dashed line indicates the average methylation level of the ICM (40.0%). Statistical significance was calculated by unpaired *t*-test. (D) Genome browser visualization of DNA methylation patterns along a segment of chromosome 10 (116,000–124,000 kb) in oocyte, blastocyst, ICM, primed PSCs, and naïve PSCs cultured in LAY (P25, P40), LADY (P25, P40), LUDY (P25, P40), LKPY (P25, P40), t2iLGö, 5iLAF, PXGL, 4CL, and HENSM. Methylation is displayed per CpG site, with bar heights reflecting methylation percentages. (E) Heatmap showing average methylation levels in oocyte-specific differentially methylated regions (DMRs) across different developmental stages, reset naïve PSCs in our four culture conditions, and in comparison, to published PSC datasets. (F) Genome browser tracks detailing DNA methylation at imprinting control regions (ICRs) in samples including oocyte, blastocyst, primed PSCs, and naïve PSCs reset in t2iLGö, 5iLAF, LAY (range 0–50), LADY (range 0–50), LUDY (range 0–50), LKPY (range 0–50), PXGL, 4CL, and HENSM. Methylation is shown per CpG site, with bar heights indicating methylation percentages. (G) Quantitative analysis of *DNMT3B* gene expression in primed PSCs and naïve PSCs cultured in LAY, LADY, LUPY, LKPY, and TJ-1# 5iLAF naïve PSCs (*n* > 2). Error bars indicate mean ± SD. Statistical significance was calculated by One-way ANOVA with GraphPad Prism. (H) Western blot image for DNMT3B and TUBLIN (loading control) protein levels in reset naïve PSCs cultured in LAY, LADY, LUPY, and LKPY conditions, TJ-1# primed PSCs, and TJ-1# 5iLAF naïve PSCs.

To systematically assess the four culture conditions in naïve pluripotency derivation and maintenance, we acquired transcriptome data through RNA sequencing (RNA-seq) of replicated samples from both prolong-cultured cells and reset cells under these four conditions, which were integrated with published data (refer to Methods) from cells reset and cultured with 5iLAF (Theunissen et al., 2014), t2iLGö (Takashima et al., 2014), PXGL (Bredenkamp et al., 2019; Guo et al., 2017), HENSM (Bayerl et al., 2021), 4CL (Mazid et al., 2022), and a variety of conventional PSCs obtained from publicly available resources as well as human embryonic lineages (Petropoulos et al., 2016; Stirparo et al., 2018; Yan et al., 2013) and our own studies (Table S2). Principal component analysis (PCA) showed that our prolong-cultured cells and reset cells cluster closely with nearly all published naïve PSCs, except for those cultured in HENSM, while distinguished from published primed PSCs (Fig. 3A). Further characterization through 3D PCA revealed that cells under these four culture conditions displayed great similarity to the transcriptome state of ICM *in vivo* (Fig. S4A). Similar to those existing naïve culture systems, naïve pluripotency-related genes, including *DPPA5*, *FGF4*, *KLF17*, *KLF5*, *SUSD2*, and *TFCP2L1* were highly upregulated in our novel naïve conditions relative to conventional primed PSCs (Figs. 3B and S4B; Table S2).

We also performed Kyoto Encyclopedia of Genes and Genomes analysis by comparing gene sets derived from differentially expressed genes in reset naïve PSCs cultured in these four conditions compared with primed PSCs. The analysis revealed significant enrichment in functional categories primarily associated with signaling pathways that regulate the pluripotency of stem cells (Fig. S4C). Additionally, when comparing the transcriptome of primed PSCs with the Gene Ontology (GO) database, we found that cells cultured in LAY media, but not in LADY, LUDY, or LKPY media, demonstrated more specific enrichment in profiles related to blastocyst development and cell differentiation involved in embryonic placenta development. Notably, all four novel culture conditions show significant GO enrichment in DNA methylation (Fig. S4D).

### Genome-wide DNA methylation status

To investigate the DNA methylation landscape during long-term culture, we conducted whole-genome bisulfite sequencing (WGBS) on reset PSCs derived and maintained in LAY, LADY, LUDY, and LKPY culture systems at Passages 25 and 40. We also included TJ-1# 5iLAF naïve PSCs at Passage 25 and TJ-1# primed PSCs for analysis. Their methylation profiles were compared with established datasets for naïve PSCs cultivated under various conditions as well as primed PSCs from previous studies (Table S3). Our analysis revealed that primed PSCs exhibited uniformly high DNA methylation levels (86.6%), mirroring post-implantation embryos (82.8%) *in vivo* (Fig. 3C) (Guo et al., 2014; Okae et al., 2014). In contrast, DNA methylation in naïve PSCs showed significant variation under different culture conditions, ranging from 29.3% to 67.2%, underscoring distinct influences of different culture media on DNA methylation (Figs. 3C and S5A; Table S3). Most published naïve PSCs, along with our reset PSCs, demonstrated considerably lower DNA methylation compared to primed PSCs, except those cultured in 4CL and HENSM conditions. Notably, methylation levels in our four novel culture conditions (42.5%–50.5%) were higher than those observed in t2iLGö and 5iLAF at Passage 25, comparable to PXGL, and closely approximate the methylation state of ICM (Guo et al., 2014) during the pre-implantation stage *in vivo* (Figs. 3C and S5A; Table S3). Upon extended culture to Passage 40, a slight decrease in genome-wide DNA methylation levels was observed in LADY, LUDY, and LKPY cell lines. However, the overall methylation levels remained consistently higher than those in 5iLAF condition. Importantly, cells cultured in LAY maintained a DNA methylation level of ~50%, with minimal changes during extended culture, exhibiting the stability of cells under this culture system (Fig. 3C; Table S3).

We also observed that in our four reset PSCs, the CpG methylation patterns at various genomic regions, including the transcription start site, gene bodies, and specific genomic regions such as the 3′UTRs, 5′UTRs, enhancers, promoters, CpG islands, introns, exons, and intergenic regions, were higher compared to those in t2iLGö- and 5iLAF-cultured naïve PSCs. However, these patterns were lower than those observed in naïve PSCs cultured in 4CL, and HENSM, as well as in primed PSCs (Fig. S5B and S5C).

Further examination into DNA methylation profiles revealed that naïve PSCs derived under LAY, LADY, LUDY, and LKPY conditions exhibited a correlation with the ICM cells (Fig. S6A). These cells converged towards a methylation pattern reminiscent of the pre-implantation human blastocyst and ICM cells (Fig. 3D). We also investigated the differentially methylated regions (DMRs), including imprinting control regions for all known imprinted regions, between oocyte and sperm, identifying DNA regions corresponding to oocyte- and sperm-specific DMRs (Zhu et al., 2018). Notably, DMR distribution in LAY-derived naïve PSCs more closely resembled that of ICM cells at Passage 40 (Figs. 3E and S6B; Table S3). Intriguingly, our developed LAY culture systems demonstrated enhanced maintenance of methylation at several imprinted loci (Court et al., 2014; Jima et al., 2022), such as *FAM50B*, *MIR512-1*, *SNU13*, and *GLIS3*. This was in contrast to previously published culture systems where naïve PSCs displayed an almost complete loss of methylation at these stable imprints as reported previously (Figs. 3F and S6C). Collectively, our developed culture conditions, particularly the LAY system, mitigated the DNA hypomethylation observed in existing naïve culture systems primarily caused by prolonged MEKi exposure, and partially restored the methylation patterns at imprinted loci (Figs. 3F and S6C) (Pastor et al., 2016).

Motivated by both GO enrichments from our RNA-seq results and improved methylation status from our WGBS data, we further analyzed the expression of DNA methyltransferases and their cofactors (Fig. S6D and S6E). Notably, our results indicate that our developed culture systems could enhance the expression of DNMT3B, a DNA methyltransferase functioning in methylation maintenance, at both RNA and protein levels, compared to TJ-1# 5iLAF naïve PSCs (Figs. 3G, 3H and S6F).

### Methylation status of transposable elements

**Figure 4.**
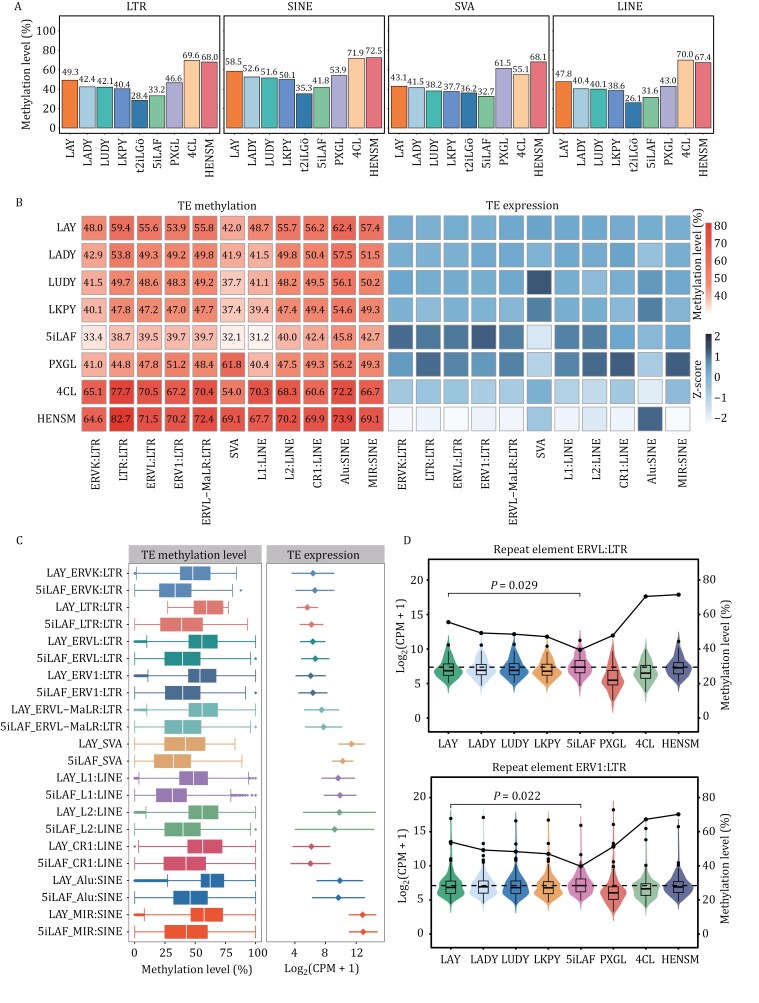
The levels of methylation and expression of transposable elements (TEs) in our optimized culture system. (A) Average methylation levels of transposable elements (TEs) in human naïve PSCs cultured in different conditions. LTR, long terminal repeats; SINE, short interspersed nuclear elements; SVA, SINE-VNTR-Alu; LINE, long interspersed nuclear elements. (B) Heatmaps illustrating the average methylation levels (left) and expression patterns (right) of TE subfamilies in naïve PSCs cultured in LAY, LADY, LUDY, LKPY, 5iLAF, PXGL, 4CL, and HENSM. (C) Methylation (left) and expression (right) profiles of LAY and 5iLAF across representative TE subfamilies. Error bars indicate mean ± SD of transposable subfamilies. (D) Violin plots depicting the expression levels of ERVL (top) and ERV1 (bottom) in naïve PSCs cultured in LAY, LADY, LUDY, LKPY, 5iLAF, PXGL, 4CL, and HENSM (left *y-*axis), with line plots showing the average methylation levels in same samples (right *y*-axis). Statistical significance was calculated by the Wilcoxon rank-sum test. The dashed line indicates the average expression of the 5iLAF.

Transposable elements (TEs), which predominantly exist as interspersed, multi-copy repeats across the genome, constitute approximately half the human genome (Liao et al., 2023). Previous studies have underscored the crucial role of DNA methylation in the extensive transcriptional silencing of TEs, with DNA hypomethylation leading to genomic instability through the activation of retrotransposons (Ohtani et al., 2018; Switzer et al., 2022). In this context, we systematically analyzed the DNA methylation levels of TEs in the aforementioned cell lines. Notably, the reset naïve PSCs derived in LAY, LADY, LUDY, and LKPY conditions displayed elevated methylation levels in LTR, SINE, SVA, and LINE elements compared to t2iLGö and 5iLAF naïve cells (Fig. 4A). Particularly, both the overall and TE-specific methylation were higher in naïve PSCs derived in LAY condition among the four culture conditions (Figs. 3C and 4A). We further explored the expression of various TE subfamilies in naïve PSCs derived in various culture conditions. We observed a decrease in transcriptional activity across various TE subfamilies, correlating with their increased DNA methylation in our developed culture systems compared with 5iLAF (Fig. 4B–D). Taken together, our findings suggest that suppression ofTE transcription activities by DNA CpG methylation is not only consistent with long-term accommodation in the host genome, but also present in the effect of culture conditions on naïve cells (Meng et al., 2015; Soto-Palma et al., 2022; Zhou et al., 2020).

### X chromosome status of LAY reset cells

**Figure 5.**
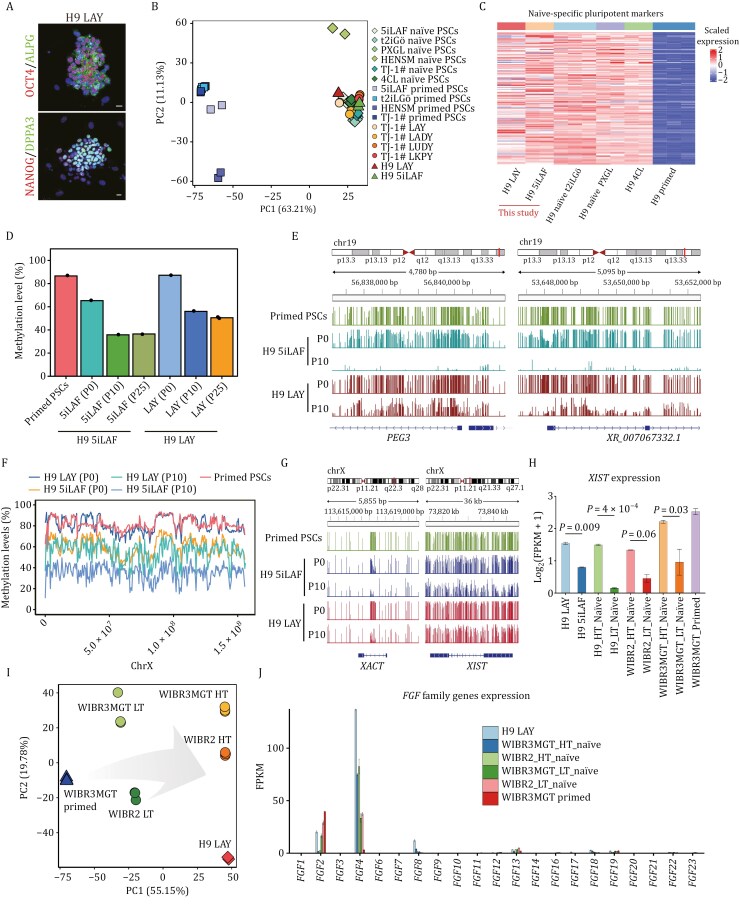
H9 LAY naïve PSCs exhibit improved global DNA methylation and active X chromosome status. (A) Immunoﬂuorescence microscopy showing the expression of naïve pluripotency markers in H9 reset naïve PSC under the LAY condition. Scale bars, 20 µm. (B) PCA of the transcriptional profiles from four TJ-1# and two H9 reset cultures (*n* = 2), compared with naïve and primed PSCs from published datasets. (C) Heatmap showing the expression of naïve-specific pluripotency markers in H9 LAY reset PSCs (*n* = 2), H9 5iLAF naïve PSCs (*n* = 2), H9 t2iLGö naïve PSCs (*n* = 3), H9 PXGL naïve PSCs (*n* = 2), H9 4CL naïve PSCs (*n* = 2), and H9 primed PSCs (*n* = 3). (D) Bar plot illustrating the average DNA methylation levels in LAY naïve PSCs (Passages 0, 10, and 25), 5iLAF naïve PSCs (Passages 0, 10, and 25), and primed PSCs. (E) Genome browser tracks detailing DNA methylation at imprinting control regions (ICRs) in samples including primed PSCs, H9 LAY (Passages 0 and 10), and H9 5iLAF (Passages 0 and 10) naïve PSCs. Methylation is shown per CpG site, with bar heights indicating methylation percentages. (F) X chromosome-wide average DNA methylation levels in the aforementioned samples by sliding window analysis. Window size: 0.5M bp. (G) Genome browser tracks detailing DNA methylation of *XACT* and *XIST* in the above samples. Methylation is shown per CpG site, with bar heights indicating methylation percentages. (H) Bar plot showing the expression level of *XIST* in H9 LAY naïve, H9 5iLAF naïve, WIBR3MGT primed, and HT and LT naive hESCs derived from H9, WIBR2, and WIBR3MGT. Statistical significance was calculated by unpaired *t*-test. (I) PCA of the transcriptional profiles in H9 LAY naïve, WIBR3MGT primed, and HT and LT naïve hESCs derived from H9, WIBR2, and WIBR3MGT. (J) Expression of *FGF* family genes quantified using average FPKM from RNA-seq data for the above samples.

Given the performance of the LAY system in terms of genome-wide DNA methylation levels, we further validated its applicability and effectiveness in the conventional H9 ESC line. Under LAY culture condition, H9 reset naïve ESCs were successfully established and exhibited robust expression of key naïve pluripotency markers, including NANOG, DPPA3, OCT4, and ALPG (Fig. 5A). PCA revealed that our H9 reset cells clustered closely with nearly all published naïve PSCs, as well as TJ-1# reset cells (Fig. 5B). Similar to naïve PSCs derived under other conditions, the H9 LAY naïve ESCs showed high expression of core naïve pluripotency genes (Fig. 5C).

WGBS analysis of H9 LAY naïve ESCs demonstrated higher global DNA methylation levels compared to H9 5iLAF naïve PSCs at both Passage 0 and Passage 10 (Fig. 5D). Notably, consistent with observations in TJ-1# reset cells, the H9 LAY ESCs maintained genome-wide DNA methylation levels around 50% and preserved DNA methylation at several imprinted loci, including *PEG3*, *MIR512-1*, *GRB10*, *GLIS3*, and *SNU13* (Figs. 5E, S7A, and S7B), suggesting the potential advantage in maintaining epigenetic stability.

Considering the critical role of DNA methylation in X chromosome inactivation (XCI), we analyzed the overall DNA methylation levels of the X chromosome in H9 LAY and H9 5iLAF cells at Passage 10 (P10), observing higher X chromosome-wide DNA methylation levels in H9 LAY cells compared to H9 5iLAF (P10) cells (Fig. 5F). We also quantified the DNA methylation levels of specific X-linked genes, including *XACT*, *XIST*, and *ATRX*, as well as *MECP2* (Figs. 5G, S7C, and S7D). Despite the overall increased DNA methylation levels in H9 LAY (P10) cells compared to H9 5iLAF (P10) cells, no significant differences were detected at these specific loci between the two conditions.

To assess XCI status, we compared H9 LAY cells to the X-linked dual reporter-engineered cell lines described previously, in which they identified two distinct populations in 5iLAF naïve ESCs: HT cells (characterized by high expression of naïve-specific markers, two active X chromosomes, and bi-allelic expression of *XIST*) and LT cells (exhibiting low expression of naïve-specific markers, an intermediate state between naïve and primed pluripotency, two active X chromosomes, and mono-allelic expression of *XIST*) (An et al., 2020). RNA-seq analysis revealed that *XIST* expression levels were higher in H9 LAY cells than in H9 5iLAF cells, closely resembling HT cells from WIBR3MGT, WIBR2MGT, and H9 (CRA002553) (Fig. 5H). PCA further confirmed that H9 LAY cells clustered with HT cells along the primary axis of variation (PC1, 55.15%) (Fig. 5I), suggesting similarities to HT cells. H9 LAY cells also displayed high *FGF4* and low *FGF2* expression (Fig. 5J), consistent with the reported expression pattern of HT cells, which displayed high *FGF4* and low *FGF2* levels, in contrast to primed PSCs that exhibited the opposite pattern. Collectively, our results demonstrate that H9 LAY cells share transcriptomic and DNA methylation features with HT cells, corresponding to a state where the X chromosome remains active.

### Improved genomic stability of LAY naïve PSCs

**Figure 6.**
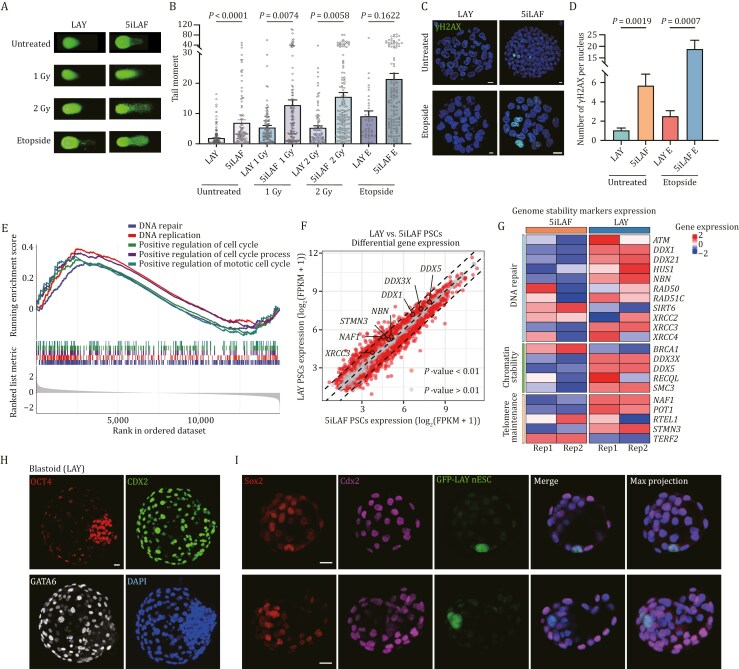
Genomic stability and development potential of LAY naïve PSCs. (A) Representative photographs from comet assays to assess DNA damage in LAY- and 5iLAF-cultured naïve PSCs exposed to untreated, X-ray (1 Gy and 2 Gy) and Etopside (2.5 μmol/L 2 h). (B) Average tail moments from comet assays to assess DNA damage in LAY- and 5iLAF-cultured naïve PSCs exposed to untreated, X-ray (1 Gy and 2 Gy) and Etopside (2.5 μmol/L 2 h). At least 20 cells were counted for each group, with error bars indicating mean ± SEM. Statistical significance was calculated by One-way ANOVA with GraphPad Prism. (C) Immunoﬂuorescence imaging of naïve PSCs cultured in LAY and 5iLAF stained with anti-γH2AX antibodies and DAPI exposed to untreated and Etopside (2.5 μmol/L 2 hours). Scale bars, 10 µm. (D) The average number of γH2AX foci per nucleus (*n* > 40). Error bars indicate mean ± SEM of different cells. Statistical significance was calculated by one-way ANOVA with GraphPad Prism. (E) Gene Set Enrichment Analysis (GSEA) showing the enriched Gene Ontology (GO) terms among genes upregulated in LAY PSCs compared to 5iLAF PSCs. (F) Scatter plot showing differential gene expression between LAY PSCs and 5iLAF PSCs. The *x*- and *y*-axes represent the expression levels of genes in 5iLAF and LAY PSCs, respectively. Each dot represents a gene. Red dots indicate *P-*value <0.01; gray dots indicate *P-*value >0.01. (G) Heatmap showing the expression levels of genomic stability-related genes in LAY- and 5iLAF-cultured naïve PSCs (*n* = 2). (H) Representative co-immunostaining images of OCT4, CDX2, GATA6, and DAPI in the blastoid derived from naïve PSCs cultured in LAY. Scale bar, 50 µm. (I) Representative co-immunostaining images of Sox2, Cdx2, and GFP signals in LAY nESC-aggregated chimeric embryos at the blastocyst stage. Scale bars, 20 µm.

Genome-wide DNA hypomethylation is a well-documented hallmark of genomic instability across various malignancies (Cadieux et al., 2006; Deniz et al., 2019). Building on previous reports that reduced MEK inhibition preserves genomic stability in human naïve ESCs (Di Stefano et al., 2018), we further investigated the influence of the culture system on genomic stability in naïve PSCs. We performed alkaline comet assays on LAY- and 5iLAF-cultured naïve PSCs at the same passage. The results demonstrated that cells cultured in LAY medium exhibited significantly fewer DNA breaks and enhanced genomic stability compared to those in 5iLAF medium (Fig. 6A and 6B) (Sun et al., 2023). Immunostaining for Phospho Histone H2A.X further corroborated these findings, showing a reduced accumulation of DNA double-strand breaks in LAY cells compared to 5iLAF cells (Fig. 6C and 6D). Moreover, 5iLAF naïve PSCs displayed marked sensitivity to DNA damage induced by either X-ray irradiation (at doses of 1 or 2 Gy) or a 2-hour Etoposide treatment, while LAY naïve cells showed significantly enhanced resistance to these genotoxic stresses (Fig. 6A and 6B). These results collectively underscore the superior genomic integrity and damage resilience of naïve PSCs cultured in LAY medium.

Differential gene expression analysis and Gene Set Enrichment Analysis comparing the LAY and 5iLAF PSCs revealed significant enrichment of pathways associated with genomic stability, including DNA repair, DNA replication, and the cell cycle, in the LAY cell line (Fig. 6E). Additionally, several key genomic stability-related genes, such as *DDX5* and *DDX3X*, were significantly upregulated in the LAY cell line (*P-*value < 0.01) (Fig. 6F). To gain deeper insight, we compiled a list of genes implicated in genomic stability based on previous studies (Adams et al., 2024; Bonilla et al., 2020; Cargill et al., 2021; Chen et al., 2020, 2023; Fu et al., 2023; Huang et al., 2023; Li et al., 2020; Xu et al., 2001), including genes involved in DNA repair (e.g., *ATM*, *DDX1*, *DDX21*, *HUS1*, *NBN*, *RAD50*, *RAD51C*, *SIRT6*, *XRCC2*, *XRCC3*, *XRCC4*), chromatin stability (e.g., *BRCA1*, *DDX3X*, *DDX5*, *RECQL*, *SMC3*), and telomere maintenance (e.g., *NAF1*, *POT1*, *RTEL1*, *STMN3*, *TERF2*) (Fig. 6G). Comparative analysis revealed that most of these genes were upregulated in the LAY system, further supporting enhanced genomic stability in cells cultured in LAY system.

To assess the lineage differentiation potential of naïve PSCs cultured in LAY and 5iLAF media over extended passaging (beyond 50 passages), we successfully generated blastoids from both LAY- and 5iLAF-cultured naïve ESCs at early (P25) and late passages (P50) through sequential treatments with enhanced trophoblast differentiation medium (eTDM) and enhanced hypoblast differentiation medium (eHDM) (Fig. S8A) (Yu et al., 2021, 2023). Comparative analyses of blastoids from P25 and P50 cells in both conditions revealed no significant differences in morphological architecture, cavitation efficiency, and overall diameter (Fig. S8A–C). Immunofluorescence staining identified the presence of OCT4-positive cells within the ICM, CDX2-positive cells located in the outer cell layer surrounding OCT4-positive cells, and GATA6-positive cells in the surface layer of inner populations and the outer enclosing cell layer (Fig. 6H), representing epiblast, trophectoderm, and hypoblast compartments, respectively.

We also evaluated the developmental competence of LAY and 5iLAF naïve PSCs in developing mouse embryos. GFP-labeled LAY or 5iLAF naïve cells were aggregated with mouse morula embryos. Immunostaining of the chimeric blastocysts revealed GFP and Sox2 double-positive cells within the inner cell mass (ICM) (Figs. 6I and S8D). Additionally, GFP signal was observed in the trophectoderm (TE) of the chimeric blastocysts, marked by Cdx2 expression (Fig. 6I), indicating that LAY cells, similar to 5iLAF cells, contributed to both embryonic and extra-embryonic lineages.

Taken together, these results underscore the robust lineage differentiation potential of naïve PSCs derived in our LAY media.

## Discussion

In this study, utilizing a previously established bifluorescence reporter and the npn transition system, we pinpointed seven chemicals that promote human naïve pluripotency through high-throughput screening, and subsequently developed four effective media for resetting and maintaining human naïve PSCs with improved genome-wide DNA methylation, closely resembling *in vivo* ICM cells, and enhanced genomic stability. Our research optimized culture conditions for further applications of human naïve PSCs.

Previous studies have reported that suppression of FGFR either by the FGFR1/2/3/4 inhibitor Debio-1347 (Zoligratinib) or the selective FGFR inhibitor AZD4547 (ABSK091) can modulate the MEK-ERK signaling pathway by affecting p-ERK expression (Nakanishi et al., 2015; Zhao et al., 2017). Among the four culture conditions we developed, Debio-1347 was included in the LADY and LUDY systems, while ABSK091 was contained in the LKPY condition. We observed a decline in DNA methylation levels of cells cultured in LADY, LUDY, and LKPY conditions to below 40% during extended culture, with LUDY, which contained both FGF and ERK inhibitors, showing the most pronounced DNA methylation loss. These results align with previous studies, suggesting that prolonged exposure of naïve PSCs to MEK pathway inhibitors, particularly FGF and ERK inhibitors, leads to a progressive decline in DNA methylation levels over time.

Notably, the LAY system distinguishes itself among those existing culture systems, showcasing that the addition of a single chemical compound, the ATP-competitive JAK2 inhibitor AZD1480, to the naïve base culture with LIF and a ROCK inhibitor, is sufficient for the acquisition and stable maintenance of naïve pluripotency, demonstrating superior genomic stability and more stable genome-wide DNA methylation patterns during extended culture, compared to the other three systems. This highlights the importance of finely-tuned inhibitory pathways in the culture system for optimal outcomes. Moreover, this finding coincides with the previous proposal that minimal MAPK activity is beneficial for the preservation of both robust growth potential and genomic stability in naïve PSCs (Di Stefano et al., 2018).

Our systematic comparison of DNA methylation status across various naïve culture systems indicates that not only pluripotency outcomes but also epigenetic characteristics and genomic stability should be considered when evaluating naïve induction strategies. Further investigation into DNA methylation patterns suggested a potential association with genomic stability, enriching our understanding of naïve pluripotency. While the culture systems we developed mitigate genome-wide DNA hypomethylation and loss of imprints commonly associated with prolonged use of the MEK inhibitor PD0325901, complete restoration of DNA methylation levels at imprinted loci to match those of *in vivo* ICM cells remains a challenge. Future efforts are needed to further refine these culture systems to accurately replicate the methylation patterns and levels of imprinted loci within pre-implantation embryos *in vivo*.

## Supplementary data

Supplementary data is available at *Protein & Cell* online https://doi.org/10.1093/procel/pwaf053.

pwaf053_suppl_Supplementary_Figures_S1-S8

pwaf053_suppl_Supplementary_Table_S1

pwaf053_suppl_Supplementary_Table_S2

pwaf053_suppl_Supplementary_Table_S3

## Data Availability

The bulk RNA-seq datasets generated in this study are available at GEO: GSE252312. The WGBS datasets generated in this study are available at GEO: GSE252313. The accession numbers for the RNA-seq data of human embryos are E-MTAB-3929 and GSE36552. The accession numbers for the RNA-seq data of published cell lines are E-MTAB-2856, GSE59435, GSE166401, CNP0001454, GSE150772, and GSE174771. The accession numbers for the WGBS data are GSE49828, JGAS00000000006, GSE60945, GSE76970, GSE90168, CNP0001454, and GSE142812. Source data are provided in this paper. All requests for materials and data should be directed to the corresponding authors.

## Code availability

This paper does not report the original code.
