# Supplementary material for: Optimized derivation and culture system of human naïve pluripotent stem cells with enhanced DNA methylation status and genomic stability
Source: Protein Cell. 2025 Sep 9;16(10):858–72. doi: 10.1093/procel/pwaf053 (PMC12578290; doi:10.1093/procel/pwaf053)
Supplement: pwaf053_suppl_Supplementary_Figures_S1-S8 [file pwaf053_suppl_supplementary_figures_s1-s8.docx]

**Materials and methods**

**Cell culture**

Human primed PSCs (TJ-1# primed PSCs, H9) were maintained in conventional PSCs medium containing DMEM/F12 (Thermo Fisher) with 20% KnockOut SR (Thermo Fisher), 1% Nonessential amino acids (Millipore), 2 mmol/L L-Glutamine (Millipore), 1% Penicillin-Streptomycin (Millipore), and 8 ng/mL bFGF (PeproTech). The medium was changed daily, and the cells were passaged by cutting colonies into small, uniform squares with glass needles every 4-5 days.

Human naïve PSCs (TJ-1# 5iLAF, H9 reset) derived from the corresponding primed PSCs were cultured in 5iLAF medium consisted of N2B27 basal media supplemented with 20 ng/mL human LIF (Millipore), 10 ng/mL bFGF (PeproTech), 50 μg/mL bovine serum albumin (BSA; Sigma), 1 μmol/L PD0325901 (Selleck), 1 μmol/L IM-12 (Selleck), 0.5 μmol/L SB590885 (Selleck), 1 μmol/L WH-4-023 (Selleck), 10 μmol/L Y-27632 (Selleck), and 20 ng/mL activin A (PeproTech). N2B27 basal media include DMEM/F-12: Neurobasal (1:1) (Thermo Fisher), 1% N2 Supplement (Thermo Fisher), 2% B27 Supplement (Thermo Fisher), 0.5% KnockOut SR (Thermo Fisher), 1% Nonessential amino acids (Millipore), 2 mmol/L L-Glutamine (Millipore), 1% Penicillin-Streptomycin (Millipore), 0.1 mmol/L β-mercaptoethanol (Sigma). human naïve cells were passaged with Accutase (Sigma) every 4-5 days as previously reported (Theunissen et al., 2014).

Human PSCs lines were used by the ethical approvals obtained from the Biological Research Ethics Committee of Tongji University and were cultured on mitomycin C-inactivated mouse embryonic fibroblast feeder layers in 5% CO_2_ at 37 °C. Mycoplasma tests were performed every week.

**High content screening**

We used ALPG-promoter-RFP and OCT4-ΔPE-GFP dual-fluorescence reporter naïve PSCs (Bi et al., 2020). For the first compound screening process (n = 3), 7 × 10^3^ naïve PSCs were seeded per well in a 384-well plate (Thermo Fisher, Cat# 142762) containing mitomycin C-inactivated mouse embryonic fibroblast (MEF) feeder layers and initially cultured in 5iLAF medium. On the second day, the medium was switched to the conventional PSCs medium and replaced daily thereafter. On day 4, the medium was changed to N2B27 basal media supplemented with 1 μmol/L of the screening compound (Selleck, Inhibitor Library, Cat# L1100) and refreshed daily. Controls included positive controls (5iLAF medium), standard controls (N2B27 basal media with 0.1% DMSO), and negative controls (conventional PSCs medium).

Images and data including the number of nuclei, GFP intensity, and RFP intensity were acquired on day 7 and analyzed. For second compound screening process (n = 3), different concentrations (0.1 μmol/L, 0.2 μmol/L, 0.4 μmol/L, 0.8 μmol/L, 1.6 μmol/L, 3.2 μmol/L, 6.4 μmol/L, 12.8 μmol/L, 25.6 μmol/L, 51.2 μmol/L) of 7 chemicals in N2B27 basal media were changed on day4 as with the first screening process. High content images were acquired using the CellInsight CX7 High Content Analysis Platform (Thermo Fisher, CX7A1110) with 40× objective. Images were analyzed using the free open-source software CellProfiler.

**The primed-to-naive transition**

For primed to naive state transition, 1 × 10^5^ dissociated single primed PSCs were seeded in conventional PSC medium supplemented with 10 μmol/L Y-27632 (Selleck). On the second day, the medium was switched to either 5iLAF medium or N2B27 basal media supplemented with LIF (Millipore), Y-27632 (Selleck) and corresponding chemicals and was refreshed daily thereafter. The naïve reset PSCs clones appear approximately 5-7 days into the process.

**Somatic cell reprogramming**

Human embryonic fibroblasts (HEFs) were obtained from the Clinical and Translational Research Center of Shanghai First Maternity and Infant Hospital, Tongji University and cultured in DMEM (Invitrogen) containing 10% FBS (Invitrogen) and 2 mmol/L L-Glutamine (Millipore). For naïve reprogramming, HEFs were infected with the doxycycline (Dox)-inducible, polycistronic OKMS lentiviral vector (Addgene) and ﬁrst cultured in conventional PSCs medium + Dox for 6 days, followed switching to either 5iLAF medium or novel culture conditions to generate naive clones.

**Flow cytometry**

For flow cytometry, cells were collected and washed with FACS buffer containing PBS supplemented with 2% FBS. The cells were washed and resuspended in FACS buffer after staining with APC-conjugated anti-SSEA4 (BioLegend, Cat# 330407, 1:50 dilution) or APC-conjugated anti-SUSD2 (BioLegend, Cat# 327408, clone: W5C5, 1:50 dilution) antibodies. All analyses were performed on Cytoflex S (Beckman Coulter) and MoFlo Astrios EQ cell sorter (Beckman Coulter). Flow cytometry data were processed using Flow Jo software (V10.0).

**Immunostaining staining**

For immunostaining, cells were fixed overnight with PBS (Thermo Fisher) containing 4% paraformaldehyde (Sigma) at 4 °C and permeabilized for 15 minutes in PBS containing 0.05% Triton X-100. After incubation with blocking buffer (PBS containing 4% BSA) for 30 minutes at room temperature, the cells were incubated with primary antibodies followed by secondary antibodies. The following primary antibodies used in this study were used: Rabbit anti-Nanog antibody (Cell Signaling Technology, Cat# 4903S, 1:200 dilution), Rabbit anti-ALPPL2 antibody (Abcam, Cat# ab96497, 1:200 dilution), Mouse anti-TFAP2C (Santa Cruz Biotechnology, Cat# sc-12762, 1:200 dilution), Mouse anti-DPPA3 (Santa Cruz, Cat# sc-376862, 1:100 dilution), Rabbit anti-Phospho Histone H2A.X(Ser139) antibody (Cell Signaling Technology, Cat# 9718S, 1:500 dilution), Mouse anti-OCT3/4 (C-10) antibody (Santa Cruz Biotechnology, Cat# sc-5279, 1:300 dilution), Rabbit anti-CDX2 (D11D10) antibody (Cell Signaling Technology, Cat# 12306S, 1:200 dilution), Goat anti-Human GATA6 antibody (R&D Systems, Cat# AF1700-SP, 1:200 dilution). Alexa Fluor conjugated secondary antibodies were then used: 488 Donkey Anti-Rabbit IgG (H + L) Antibody (Invitrogen, Cat# A-21206, 1:500 dilution), 594 Donkey Anti-Mouse IgG (H + L) Antibody (Thermo Fisher, Cat# A-21203, 1:500 dilution), 647 Donkey Anti-Rabbit IgG (H + L) Antibody (Thermo Fisher, Cat# A-31573, 1:500 dilution), 647 Donkey Anti-Goat IgG (H + L) Antibody (Thermo Fisher, Cat# A-21447, 1:500 dilution). Nuclei were stained with 4’,6-diamidino-2-phenylindole (Sigma, Cat# D8417, 1:1000 dilution). Images were taken using the Olympus FV3000 series (Confocal Laser Scanning Microscope).

**Karyotyping**

Naive PSCs were cultured in 6-well plates in 5iLAF or alternative media (LAY, LADY, LUDY, LKPY) for the indicated passage numbers. 10 μg/mL colchicine was added during the logarithmic phase of cell growth for 10 hours. Cells were then treated with 0.075 M KCl under hypotonic conditions, followed by fixation with a fixative solution (methanol: acetic acid = 3:1). Finally, the cells were stained using Giemsa Staining Solution (Beyotime, Cat# C0133).

**Western blotting**

Cells were lysed in RIPA buffer containing 50 mmol/L Tris pH 7.0, 150 mmol/L NaCl, 1% NP40, 0.1% SDS, 1% Sodium deoxycholate, and protease inhibitor cocktail. Proteins were separated by SDS-PAGE and transferred onto a PVDF membrane. After blocking, the membrane was incubated with primary antibodies followed by secondary antibodies, and visualized with enhanced chemiluminescence reagent. The following primary antibodies used in this study were used: Rabbit anti-DNMT3B antibody (Proteintech, Cat# 26971-1-AP, 1:1500 dilution), Mouse anti-DNMT1 antibody (Novusbio, Cat# NB100-56519, 1:2500 dilution), Mouse anti-DNMT3A [64B1446] antibody (Abcam, Cat# ab13888, 1:1000 dilution), Rabbit anti-DNMT3L [EPR18774] antibody (Abcam, Cat# ab194094, 1:1000 dilution), Rabbit anti-UHFR1 antibody (absin, Cat# abs136449, 1:1000 dilution).

**Bulk RNA-seq library generation and sequencing**

Total RNA was isolated from cells using trizol (Takara Bio, Cat# 9109). To generate RNA-sequencing libraries, KAPA stranded mRNA-Seq kit (KAPA Biosystems, Cat# KK8420) was used following the manufacturer’s instructions. Adapter ligation was performed using the TruePrep Index Kit V2 for Illumina (Vazyme). Paired-end 150 bp sequencing was subsequently performed on a Novaseq 6000 (Illumina) at Berry Genomics Corporation.

The RNA-seq sequencing reads were trimmed to remove adapters and low-quality reads using trim_galore version 0.6.6 (parameters: --j 2 --quality 20 --paired) (http://www.bioinformatics.babraham.ac.uk/projects/trim_galore/) and aligned to the human genome hg38 reference genome with HISAT2 version 2.2.0 (parameters: -p 10 --summary-file -S) (Kim et al., 2019). Gene counts were derived from the number of uniquely aligned unambiguous reads by featureCounts version 2.0.3 (Liao et al., 2014). To perform differential gene expression analysis, the expression levels were quantified as FPKM and processed using limma version 3.50.3 (Ritchie et al., 2015). The differentially expressed genes used for KEGG and GO were generated from the naïve dataset of this study and published primed datasets (Theunissen et al., 2014; Bi et al., 2020). The DEGs were determined following the cutoffs of Log2 fold-change (FC) > 1.5 (upregulated) or < -1.5 (downregulated) and adjusted *p* value < 0.05. For public RNA-seq datasets, the batch effects of the samples were removed using removeBatchEffect function in the R package limma 3.50.3.

Enrichment analysis of Gene Ontology (GO) and Kyoto Encyclopedia of Genes and Genomes (KEGG) were carried out using the clusterProfiler R package with default parameters (Yu et al., 2012). Significant GO terms and KEGG pathways were visualized with the ggplot function in the ggplot2 package in R.

Gene Set Enrichment Analysis (GSEA) was conducted using the clusterProfiler R package with default parameters (Yu et al., 2012). Significantly enriched pathways from the GSEA were visualized with the ggplot function in the ggplot2 package in R.

**WGBS library generation and sequencing**

Genomic DNA was extracted from naïve or primed cells using the Wizard Genomic DNA Purification Kit (Promega, Cat# 9fB022). Whole Genome Bisulfite conversions were performed with 50 ng of gDNA using the MethylCode Bisulfite Conversion Kit (Invitrogen, Cat# MECOV-50). WGBS libraries were generated using the EpiArt DNA Methylation Library Kit for Illumina V3 (Vazgme, Cat# NE103) following the manufacturer’s instructions. Paired-end 150-bp sequencing was performed on a NovaSeq (Illumina) platform in Berry Genomics and Novogene. The WGBS sequencing reads were trimmed to remove adapters and low-quality reads using trim_galore version 0.6.6 (parameters: --clip_R1 10 --clip_R2 10 --quality 20 --paired) and aligned to the human genome hg38 reference genome with Bismark version 0.22.3 (--bowtie2 -p 8) (Krueger and Andrews, 2011). The coverage files recording the methylation state of CpG sites were inputted into the R package methylKit version 1.20.0 (Akalin et al., 2012) for further analysis. The genome-wide average % DNA methylation of CpGs was tiled into 1 kb sliding windows and only CpG sites with at least 5‐fold read coverage were retained. Average methylation level was calculating by taking the average over all covered at least 5‐fold CpG sites. The genomic region, such as CGIs, enhancer, 3’ UTRs, and 5’ UTRs were downloaded from UCSC tables with hg38 track. The DNA methylation level was calculated as the average DNA methylation levels of all CpG sites covered within the region with at least 5-fold CpG sites. The ‐15 kb upstream of the transcription start site (TSS) and 15 kb downstream of the transcription end site (TES) of each gene were divided into 100 bp windows, and the gene body range from TSS to TES was divided into 100 fractions. The average DNA methylation levels within every window or faction were calculated, and then the average value of each genomic location type was calculated to delineate the profile methylation pattern around the gene region of each sample.

For differential methylated regions identification, we systematically compared the DNA methylation levels of 1kb tiles. We assigned 1kb tiles as a specific DMR when the methylation level of these tiles is greater than 75% in one type of gamete and less than 25% in another type of gamete. Differential analysis of methylated regions was performed with the methylkit R package (settings: difference = 25, type = “all”, chunk.size = 1e+06, *q* value = 0.01).

For DNA methylation levels analysis within various transposable elements, transposable elements (TEs) were annotated from UCSC Genome Browser (RepeatMasker), such as LINEs, SINEs and LTRs and their subfamilies. The DNA methylation level was calculated as the average DNA methylation levels of all CpG sites covered within the region with at least 5-fold CpG sites.

For transposon expression profiling analysis, the RNA-seq sequencing reads were trimmed to remove adapters and low-quality reads using trim_galore version 0.6.6 (parameters: --j 2 --quality 20 --paired). The trimmed reads were mapped to human genome hg38 using STAR version 2.7.10a (--readFilesCommand zcat ‐‐outFilterMultimapNmax 100 --winAnchorMultimapNmax 200) (Dobin et al., 2013). Transposable elements (TEs) were annotated from UCSC Genome Browser (RepeatMasker). TE transcripts version 2.2.1 (Jin et al., 2015) was used to estimate TE abundances. GTF file of transposable element annotations was downloaded from https://hammelllab.labsites.cshl.edu/software/#TEtranscripts.

**Comet assay**

Cells were plated in 12-well plates and cultured for 72 hours, then treated with 2.5 μmol/L Etoposide (Sigma, Cat# E1383) for 2 hours, or exposed by 1 Gy or 2 Gy of X-ray irradiation. After treatment, the cells were allowed to recover in a 5% CO_2_ incubator at 37 °C for 2 hours. Following recovery, the cells were collected without feeder layers and resuspended in PBS at a density of 3.5 × 10^5^ cells per milliliter. The alkaline comet assay was performed according to the manufacturer’s instructions (Trevigen, Cat# 4250-050-K). DNA damage was measured in terms of tail moments using software (casplab_1.2.3b2).

**Generation of human blastoids**

For the generation of human blastoids, LAY or 5iLAF PSCs were dissociated by Accutase and counted and subjected to blastoid induction following a previously reported protocol (Yu et al., 2023). Briefly, cells were precoated with eHDM medium for 2 days, then the medium was switched to eTDM medium for 6 days. The eHDM was prepared using the following: DMEM/F-12: Neurobasal (1:1) (Thermo Fisher), 1% N2 supplement (Thermo Fisher), 2% B27 supplement (Thermo Fisher), 1% GlutaMAX (Millipore), 1% Nonessential amino acids (Millipore), 0.1 mmol/L β-mercaptoethanol (Sigma), 0.5% Penicillin–streptomycin (Millipore), 20 ng/mL bFGF (Peprotech), 20 ng/mL Activin A (Peprotech), 3 μmol/L CHIR99021 (Selleck) and CEPT cocktail (50 nmol/L Chroman 1 (MedChem Express), 5 μmol/L Emricasan (Selleck), 1× polyamine supplement (Sigma), and 0.7 μmol/L TransISRIB (Tocris)). The eTDM was prepared using the following: DMEM/F-12: Neurobasal (3:1) (Thermo Fisher), 0.25% N2 supplement (Thermo Fisher), 0.25% B27 supplement (Thermo Fisher), 0.5% GlutaMAX (Millipore), 0.5% Nonessential amino acids (Millipore), 0.1 mM β-mercaptoethanol (Sigma), 0.5% KnockOut SR (Thermo Fisher), 0.5% Penicillin–streptomycin (Millipore), 1 μmol/L PD0325901 (Selleck), 2 μmol/L A83-01 (Selleck), 0.5 μmol/L SB590885 (Selleck), 1 μmol/L WH-4-023 (Selleck), 10 ng/mL recombinant human LIF (Millipore), 0.5 μmol/L LPA (Sigma) and CEPT cocktail (50 nmol/L Chroman 1 (MedChem Express), 5 μmol/L Emricasan (Selleck), 1× polyamine supplement (Sigma), and 0.7 μmol/L TransISRIB (Tocris)).

**Chimeric embryo generation**

GFP-labeled LAY or 5iLAF naïve ESCs were dissociated into single cell using Accutase and seeded on a plate pre-coated with 0.1% gelatin (Sigma) to remove feeder cells. Aliquots of 10 - 15 single cells were meticulously transferred into the concave wells of an aggregation plate. Concurrently, embryos at the 4-cell stage were enzymatically liberated from their zona pellucida using a 20 mg/mL solution of Pronase E (Sigma, Cat# P8811). Pairs of these embryos were then co-cultured with the single cells within the depression wells. The co-cultured aggregates were maintained in G-1 PLUS medium (Vitrolife, Cat# 10128) until they developed to the blastocyst stage. Chimeric embryos were fixed for further staining.

**Statistical analyses**

For bulk RNA-seq data of the naïve PSCs, n = 2 biological replicates were obtained for each sample in alternative media (LAY, LADY, LUDY, LKPY). For WGBS data of the PSCs, biological replicates were obtained for each sample, for TJ-1# LAY (P25), TJ-1# LADY (P25), TJ-1# LUDY (P25), TJ-1# LKPY (P25), n = 2. For TJ-1# LAY (P40), TJ-1# LADY (P40), TJ-1# LUDY (P40), TJ-1# LKPY (P40), H9 LAY (P0), H9 LAY (P10), H9 5iLAF (P0), H9 5iLAF (P10), TJ-1# 5iLAF and TJ-1# Primed, n = 1.

For bar plot in Fig. 3G, 6B and 6D, statistical analysis was done with GraphPad Prism (v.9.5.1). Data of bar charts are represented as the mean ± SD or SEM. Statistical significance was calculated by One-way ANOVA. The number of replicates for each experiment is presented in the figure legends.

**Fig. S1**


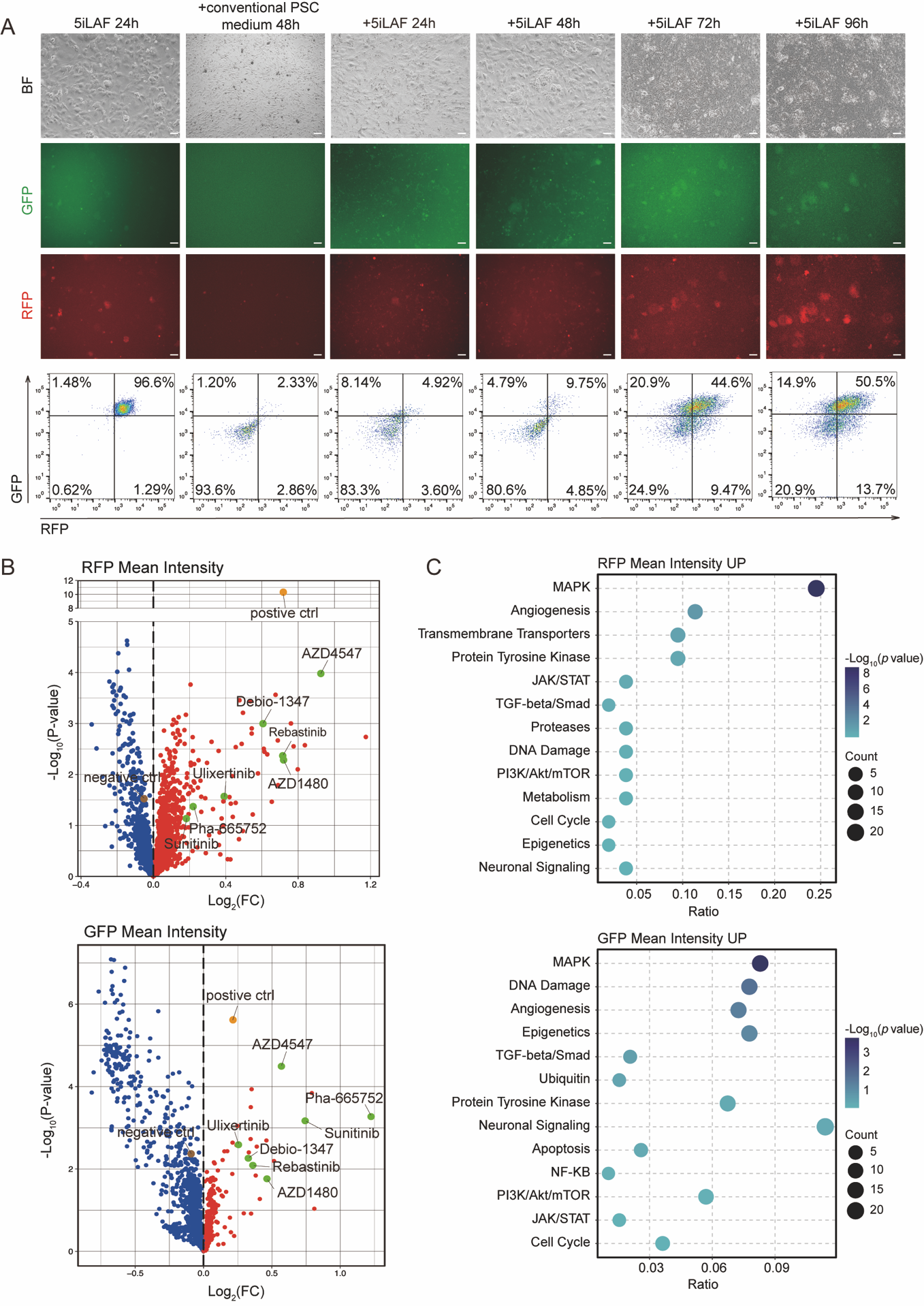


**Fig. S1. High-content screening identifies key chemicals that enhance human naïve pluripotency within a naïve-primed-naïve transition system, Related to Fig. 1.**

(A) Phase-contrast images (top), fluorescence images (middle), and flow cytometry analyses for RFP and GFP expression (bottom) in TJ-1# 5iLAF naive PSCs induced in the naïve-primed-naïve (npn) transition progress. Scale bar, 200 μm.

(B) Volcano plot showing the mean intensity of RFP (top) and GFP (bottom) across over 1600 screened chemicals. 7 significant chemicals are highlighted in both readouts.

(C) Enriched chemical ontology terms for chemicals with a fold change > 1 in RFP (top) and GFP expression (bottom). Statistical significance was calculated by hypergeometric test. Count indicates the number of chemicals with each term. Ratio refers to the proportion of chemicals in a term relative to the total number of screened chemicals.

**Fig. S2**


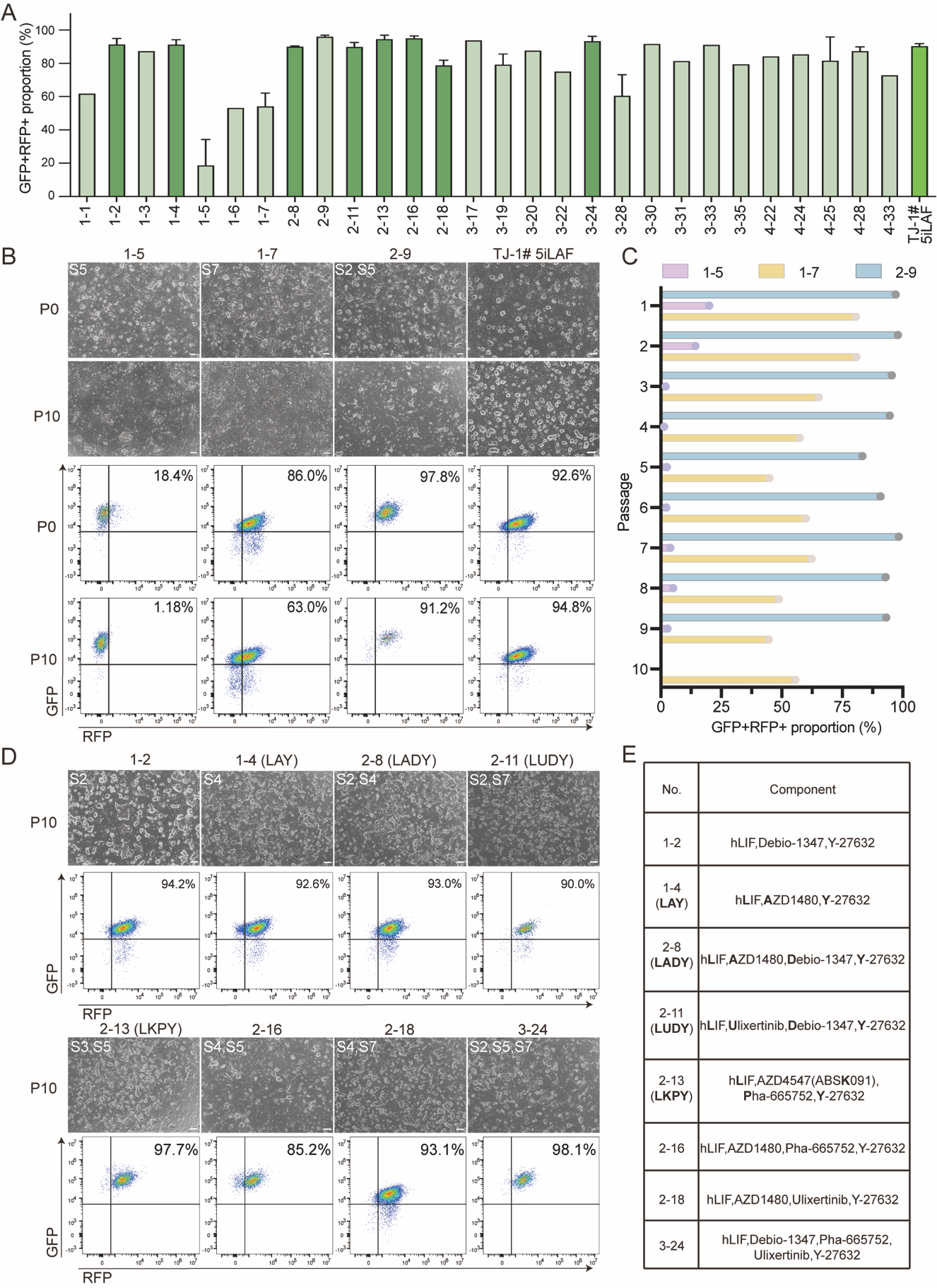


**Fig. S2. Identification of culture media efficacy for sustained growth of naïve PSCs through key chemical combinations, Related to Fig. 2.**

(A) The percentage of RFP^+^GFP^+^ TJ-1# naive PSCs, initially cultured in 5iLAF and subsequently transitioned to 28 different media via chemical combinations (Table S1), assessed up to Passage 3. Error bars indicate the mean ± SD over two or three passages. Columns for cells that survived only a single generation lack error bars. The eight culture conditions where the addition of human LIF (L) and the ROCK inhibitor Y-27632 (Y) allowed cells to maintain a high double-positive ratio and be cultured for up to 10 passages were marked in dark green, while the 5iLAF culture condition, serving as a control were labeled in bright green.

(B) Morphological (top) and fluorescence analysis for RFP and GFP signals (bottom) of TJ-1# naive PSCs initially cultured in 5iLAF and then switched to media 1-5, 1-7, and 2-9 at Passage 0 and Passage 10. Scale bar, 200 μm.

(C) Percentage of RFP^+^GFP^+^ TJ-1# naive PSCs that were derived in 5iLAF and transitioned to media 1-5, 1-7, and 2-9 across Passage 1 to Passage 10.

(D) Morphological (top) and fluorescence analysis for RFP and GFP signals (bottom) of TJ-1# naive PSCs initially cultured in 5iLAF and then switched to media 1-2, 1-4 (LAY), 2-8 (LADY), 2-11 (LUDY), 2-13 (LKPY), 2-16, 2-18, and 3-24 at Passage 10. Scale bar, 200 μm.

(E) Chemical composition of the eight media in Fig. S2D.

**Fig. S3**

**
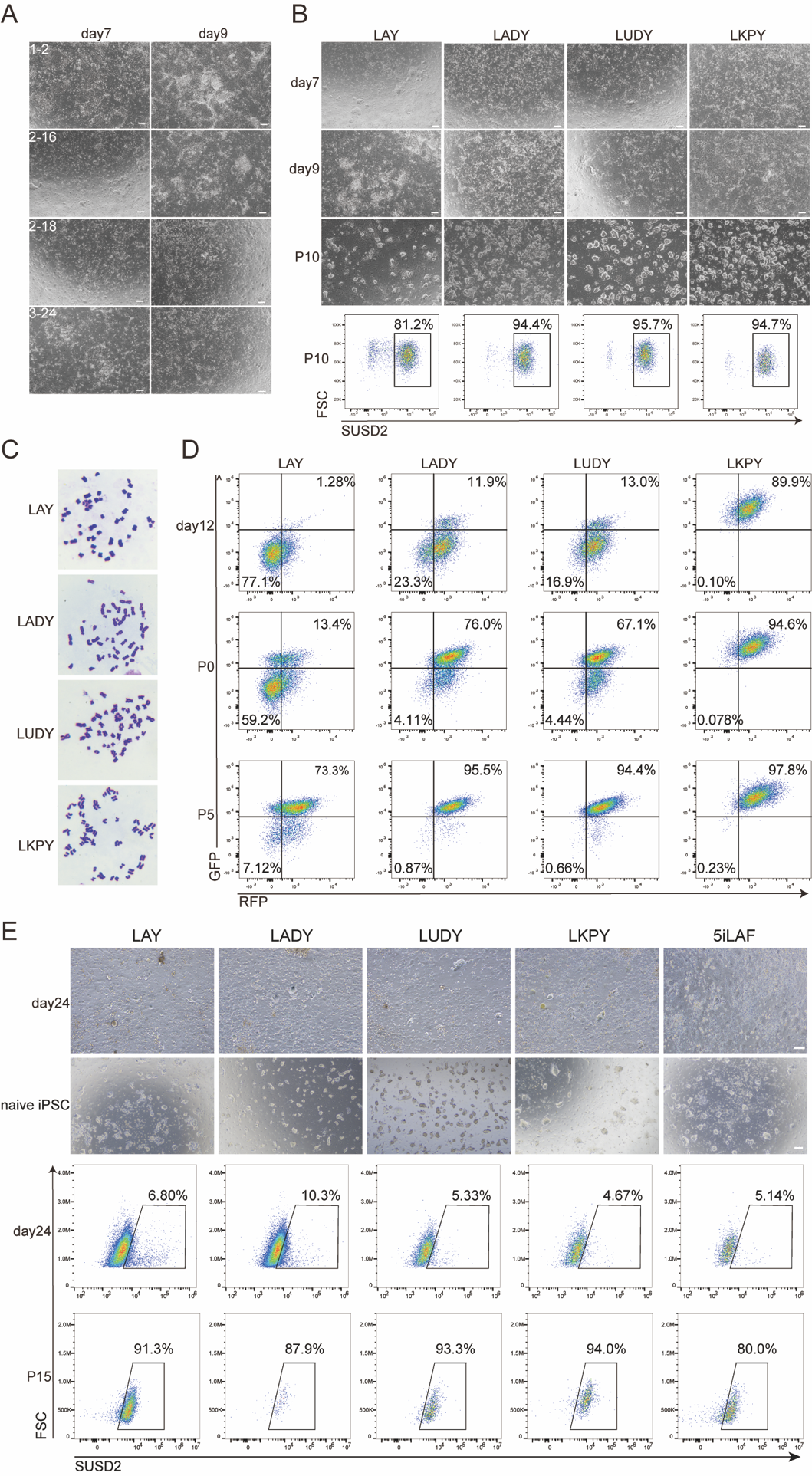
**

**Fig. S3. Identification of 4 culture media effective for the primed-to-naive resetting and naive reprogramming of somatic fibroblasts, Related to Fig.2.**

(A) Phase-contrast images showing the morphological changes of TJ-1# primed PSCs during naive resetting in culture media 1-2, 2-16, 2-18, and 3-24. Scale bar, 200 μm.

(B) Phase-contrast images of TJ-1# primed PSCs during naïve resetting process (top) and flow cytometry analyses probing for SUSD2 at Passage 10 (bottom) in culture media LAY, LADY, LUDY, and LKPY. Scale bar, 200 μm.

(C) Karyotyping images of LAY, LADY, LUDY, LKPY reset cells over Passage 25.

(D) Flow cytometry analysis for RFP and GFP expression in TJ-1# primed PSCs engineered with the bifluorescence reporter system during naïve resetting process to Passage 5 in culture media LAY, LADY, LUDY, and LKPY.

(E) Phase-contrast images of human embryonic fibroblasts on day 24 during the naïve reprogramming process (top) and the resulting naïve iPSCs (middle) under the LAY, LADY, LUDY, LKPY, and 5iLAF (control) conditions, with flow cytometry analyses for SUSD2 expression during reprogramming to Passage 15 (bottom). Scale bar, 200 μm.

**Fig. S4**

**
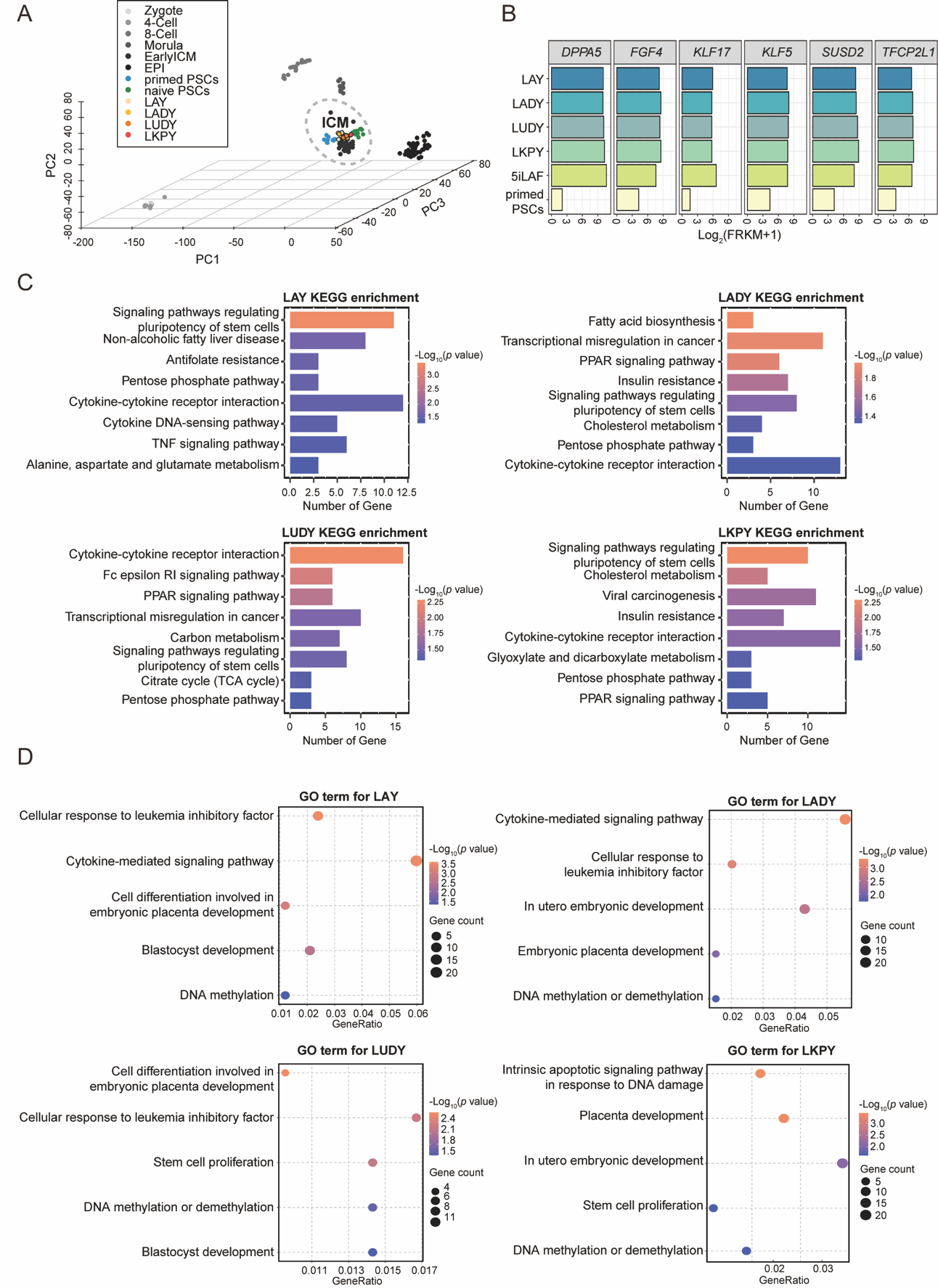
**

**Fig. S4. Transcriptional profiles of these four culture conditions for naïve pluripotency induction and maintenance, Related to Fig. 3.**

(A) Principal-component analysis (PCA) of published primed and naïve PSCs cultured in LAY, LADY, LUDY, LKPY, and human embryonic lineages at various developmental stages.

(B) Bar plot showing the expression levels of naïve-specific genes (*DPPA5*, *FGF4*, *KLF17*, *KLF15*, *SUSD2* and *TFCP2L1*) in primed PSCs and naïve PSCs cultured in LAY, LADY, LUDY, LKPY, and 5iLAF.

(C) Enriched Kyoto Encyclopedia of Genes and Genomes (KEGG) pathway analysis for gene upregulated in naïve PSCs cultured in LAY, LADY, LUDY, and LKPY conditions compared to primed PSCs. Statistical significance was calculated by hypergeometric test.

(D) Enriched Gene Ontology (GO) terms for genes that are upregulated in LAY, LADY, LUDY, and LKPY conditions compared to primed PSCs. Statistical significance was calculated by hypergeometric test.

**Fig. S5**

**
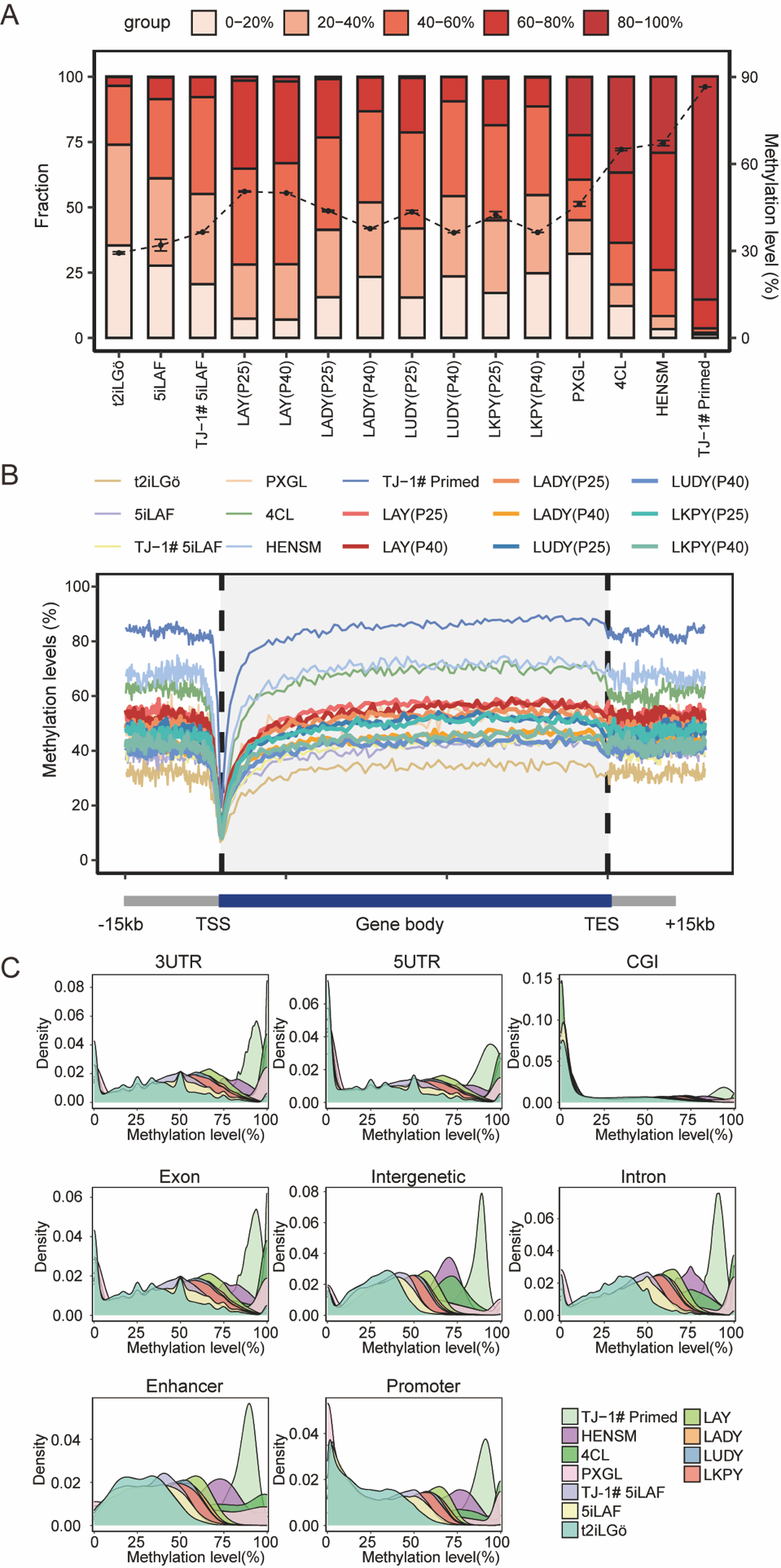
**

**Fig. S5. Comparative DNA methylation profiles across our developed and established culture conditions, Related to Fig. 3.**

(A) Histogram showing the distribution of global DNA methylation percentages (left y axis) and line plots depicting the average methylation levels (right y axis) across various primed and naïve PSC culture conditions.

(B) DNA methylation patterns along the gene bodies, extending 15 kb upstream of the transcription start sites (TSS) and 15 kb downstream of the transcription end sites (TES) of all RefSeq genes across various primed and naïve PSC culture conditions.

(C) Density distribution of DNA methylation levels in different genomic regions in PSCs under various culture conditions.

**Fig. S6**


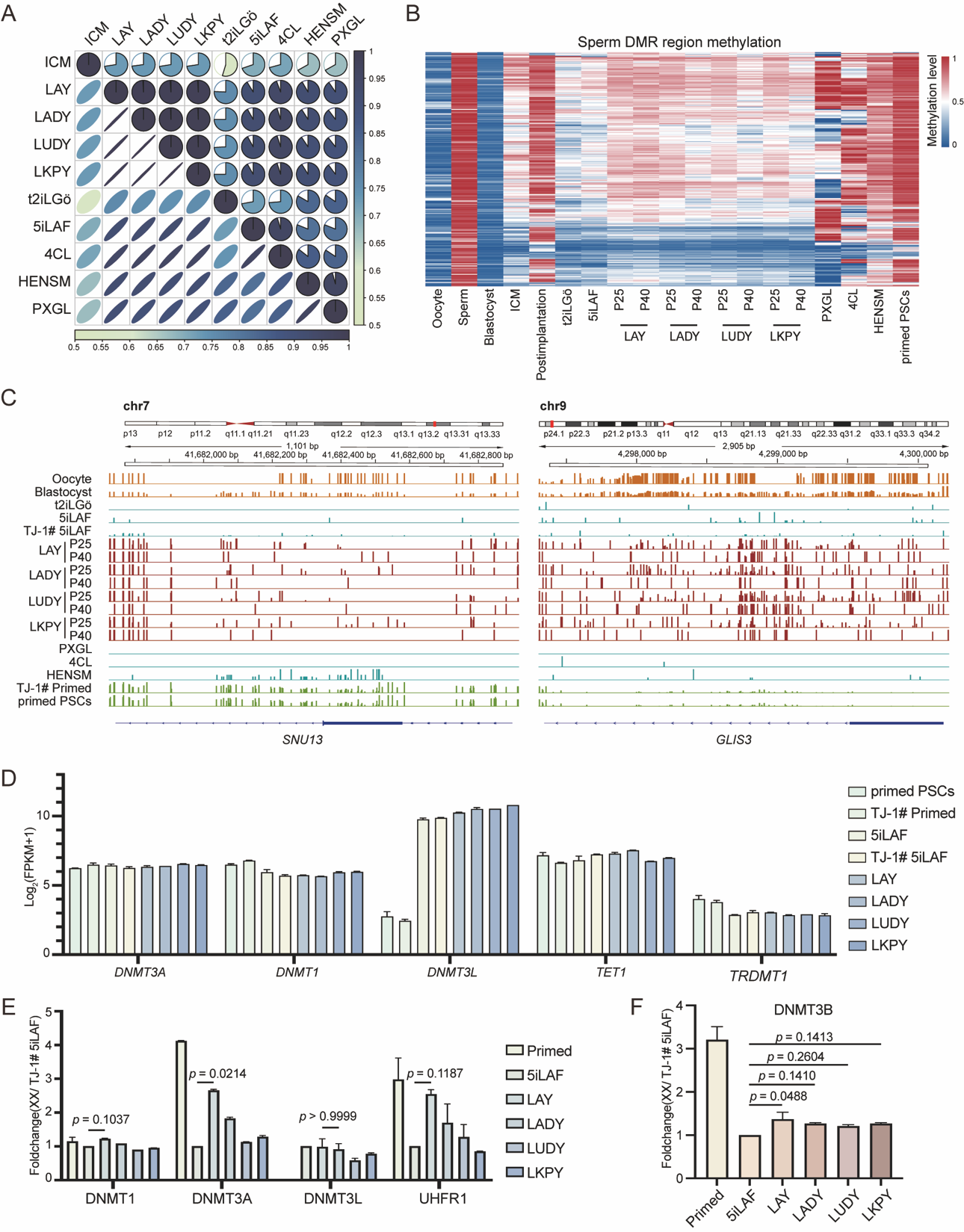


**Fig. S6. DNA methylation features across** **our developed and existing PSCs culture conditions, Related to Fig. 3.**

(A) Pearson correlation of merged methylation regions from ICM and naïve PSCs cultured in LAY, LADY, LUDY, LKPY, t2iLGö, 5iLAF, 4CL, HENSM and PXGL.

(B) Heatmap showing average methylation levels within sperm-specific DMRs across the naïve PSCs culture in our four media, different developmental stages and published PSCs.

(C) Genome browser visualization of DNA methylation patterns at specific ICRs in oocyte, blastocyst, primed PSCs, and naive PSCs cultured in t2iLGö, 5iLAF, LAY, LADY, LUDY, LKPY, PXGL, 4CL, and HENSM. Methylation is shown per CpG site, with bar heights indicating methylation percentages. For LAY, LADY, LUDY, and LKPY, the data range of *SNU13* is 0-10, and *GLIS3* is 0-10.

(D) RNA-seq analysis of *DNMT3A*, *DNMT1*, *DNMT3L*, *TET1*, and *TRDM1* expressions in primed PSCs and naïve PSCs cultured in 5iLAF, LAY, LADY, LUPY, and LKPY (n ≥ 2). Error bars indicate the mean ± SD.

(E) Quantitative analysis of western blot results for DNMT1, DNMT3A, DNMT3L, and UHFR1, using TUBULIN as the loading control, in primed PSCs and naïve PSCs cultured in LAY, LADY, LUPY, LKPY, and 5iLAF (n = 2). The y-axis indicates the fold change in protein levels relative to TJ-1# 5iLAF naïve PSCs. Error bars indicate the mean ± SEM. Statistical significance was calculated by Two-way ANOVA with GraphPad Prism.

(F) Quantitative analysis of western blot results for DMNT3B, using TUBULIN as the loading control, in primed PSCs and naïve PSCs cultured in LAY, LADY, LUPY, LKPY, and 5iLAF (n = 2). The y-axis indicates the fold change in protein levels relative to TJ-1# 5iLAF naïve PSCs. Error bars indicate the mean ± SEM. Statistical significance was calculated by One-way ANOVA with GraphPad Prism.

**Fig. S7**

**
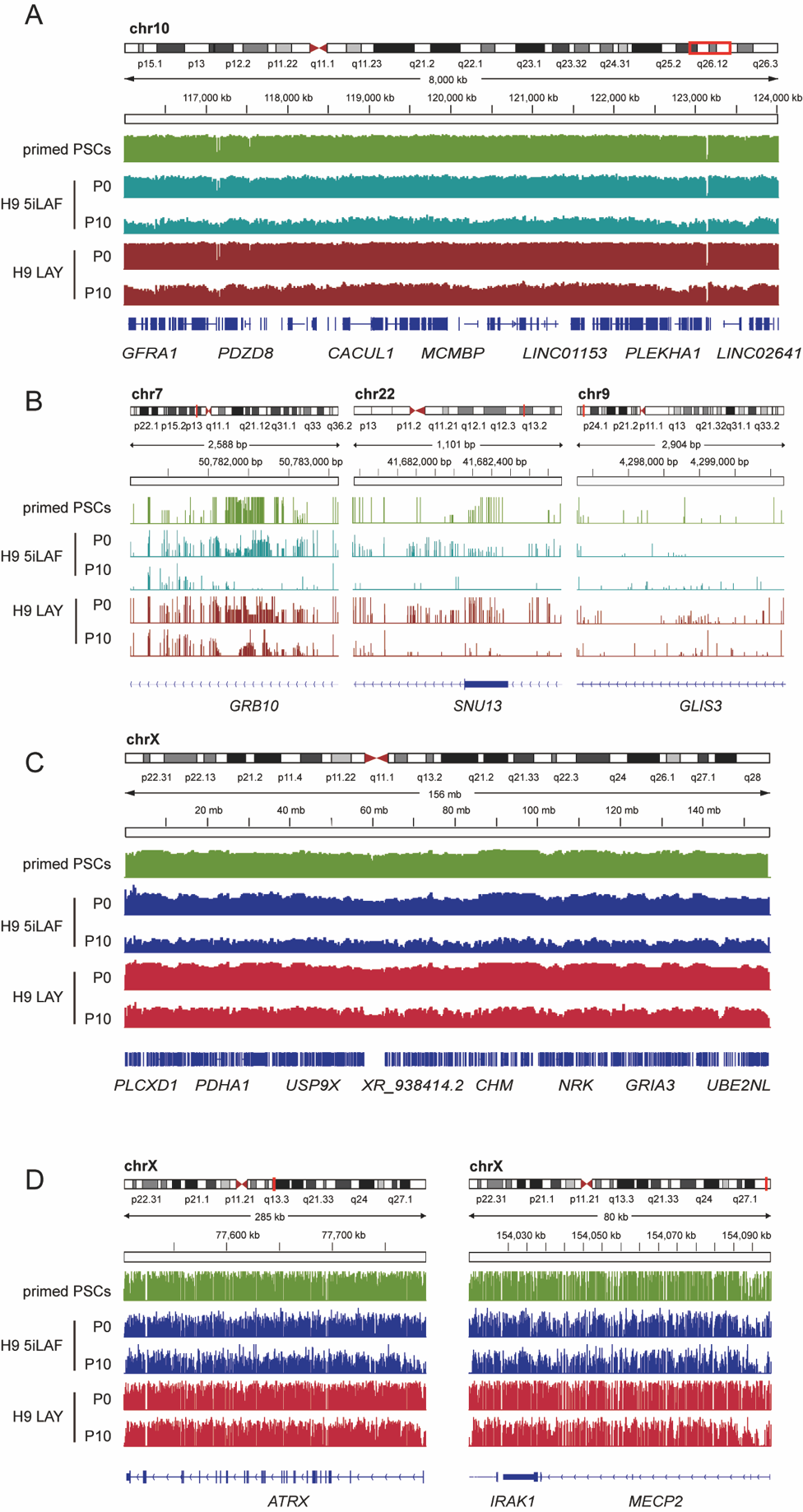
**

**Fig. S7. DNA methylation patterns in H9 LAY naïve ESCs and H9 5iLAF naïve PSCs, Related to Fig. 5.**

(A) Genome browser visualization of DNA methylation patterns along a segment of chromosome 10 (116,000 kb-124,000 kb) in H9 primed ESCs, 5iLAF naïve ESCs (Passage 0 and 10), and LAY naïve ESCs (Passage 0 and 10). Methylation is displayed per CpG site, with bar heights reflecting methylation percentages.

(B) Genome browser visualization of DNA methylation patterns at specific ICRs in H9 primed ESCs, 5iLAF naïve ESCs (Passage 0 and 10), and LAY naïve ESCs (Passage 0 and 10). Methylation is shown per CpG site, with bar heights indicating methylation percentages.

(C) Genome browser visualization of DNA methylation patterns along a segment of chromosome X in H9 primed ESCs, 5iLAF naïve ESCs (Passage 0 and 10), and LAY naïve ESCs (Passage 0 and 10). Methylation is displayed per CpG site, with bar heights reflecting methylation percentages.

(D) Genome browser tracks detailing DNA methylation of *ATRX* and *MECP2* in H9 primed ESCs, 5iLAF naïve ESCs (Passage 0 and 10), and LAY naïve ESCs (Passage 0 and 10). Methylation is shown per CpG site, with bar heights indicating methylation percentages.

**Fig. S8**


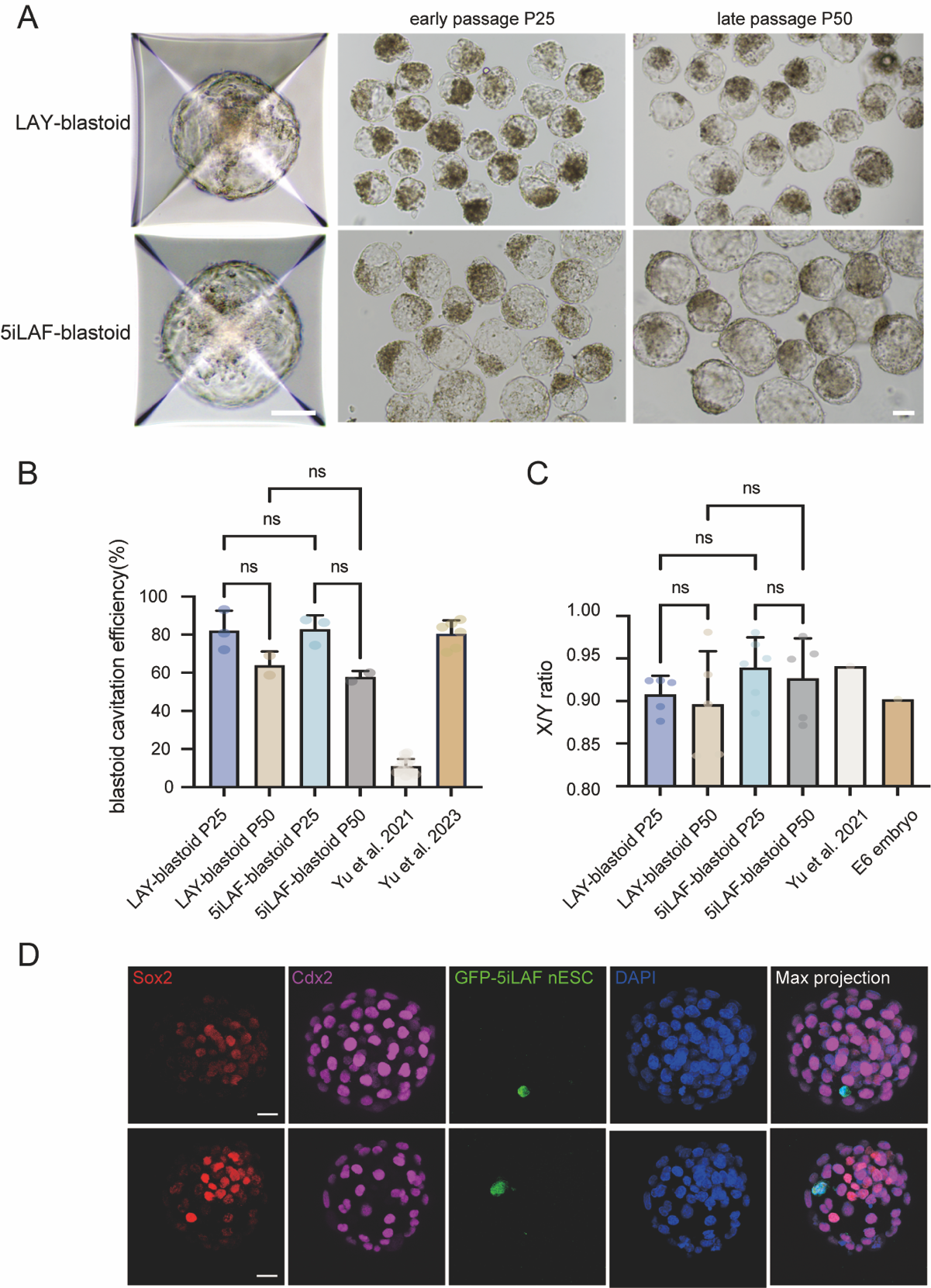


**Fig. S8. LAY naïve ESCs retain comparable differentiation competence with 5iLAF naïve ESCs, Related to Fig. 6.**

(A) Representative phase-contrast images comparing blastoids derived from early-passage (P25) and late-passage (P50) naïve ESCs cultured in LAY and 5iLAF conditions. Scale bar, 100 μm.

(B) Efficiencies of cavity-containing structure formation of blastoids shown in Fig. S8A as well as published blastoids (data from Yu et al., 2021; Yu et al., 2023). Error bars indicate mean ± SD. Statistical significance was calculated by One-way ANOVA with GraphPad Prism.

(C) Measurement of the x/y ratio of blastoids shown in Fig. S8A and published blastoids (data from Yu et al., 2021), compared to blastocysts (n = 13) (data for blastocysts from Liu et al., 2021). Error bars indicate mean ± SD. Statistical significance was calculated by One-way ANOVA with GraphPad Prism.

(D) Representative coimmunostaining images of Sox2 (red), Cdx2 (pink) and GFP signals in 5iLAF naïve ESC-aggregated chimeric embryos at the blastocyst stage. Scale bars, 20 μm.

**Table S1.** The information of combination media in our datasets. The optimal concentration of seven chemicals was utilized by high-content screening, resulting in 127 combination media, and three controls were set.

**Table S2.** The Bulk RNA-seq information for reset and prolong cultures in our datasets. Published data used in this study, including the Bulk RNA-seq datasets, the scRNA-seq datasets and the Bisulfite-Seq datasets. The FPKM information of the Bulk RNA-seq transcriptional profiles from reset and prolong cultures from our datasets, compared with naïve and primed PSCs from published datasets. Transcriptional profiles of the human naive-specific genes used in this study.

**Table S3.** The Bisulfite-Seq information for TJ-1# 5iLAF naïve PSCs, TJ-1# primed PSCs and the 4 culture conditions in our datasets. Global DNA methylation levels across different developmental stages, reset naïve PSCs in our 4 culture conditions, and in comparison, to published PSC datasets. DNA methylation levels in oocyte-specific and sperm-specific differentially methylated regions (DMRs) in the above samples.
